# Recent advances in the fluorimetric and colorimetric detection of cobalt ions

**DOI:** 10.1039/d4ra00445k

**Published:** 2024-03-25

**Authors:** Muhammad Shahbaz, Birra Dar, Shahzad Sharif, Muhammad Aqib Khurshid, Sajjad Hussain, Bilal Riaz, Maryam Musaffa, Hania Khalid, Ayoub Rashid Ch, Abia Mahboob

**Affiliations:** a Materials Chemistry Laboratory, Department of Chemistry, Government College University Lahore 5400-Pakistan mssharif@gcu.edu.pk; b School of Chemistry, Faculty of Basic Sciences and Mathematics, Minhaj Univeristy Lahore Pakistan

## Abstract

Cobalt is an essential metal to maintain several functions in the human body and is present in functional materials for numerous applications. Thus, to monitor these functions, it is necessary to develop suitable probes for the detection of cobalt. Presently, researchers are focused on designing different chemosensors for the qualitative and quantitative detection of the metal ions. Among the numerous methods devised for the identification of cobalt ions, colorimetric and fluorimetric techniques are considered the best choice due to their user-friendly nature, sensitivity, accuracy, linearity and robustness. In these techniques, the interaction of the analyte with the chemosensor leads to structural changes in the molecule, causing the emission and excitation intensities (bathochromic, hyperchromic, hypochromic, and hypsochromic) to change with a change in the concentration of the analyte. In this review, the recent advancements in the fluorimetric and colorimetric detection of cobalt ions are systematically summarized, and it is concluded that the development of chemosensors having distinctive colour changes when interacting with cobalt ions has been targeted for on-site detection. The chemosensors are grouped in various categories and their comparison and the discussion of computational studies will enable readers to have a quick overview and help in designing effective and efficient probes for the detection of cobalt in the field of chemo-sensing.

## Abstract 2

Cobalt is an essential metal to maintain several functions in the human body and is present in functional materials for numerous applications.

## Graphical abstract

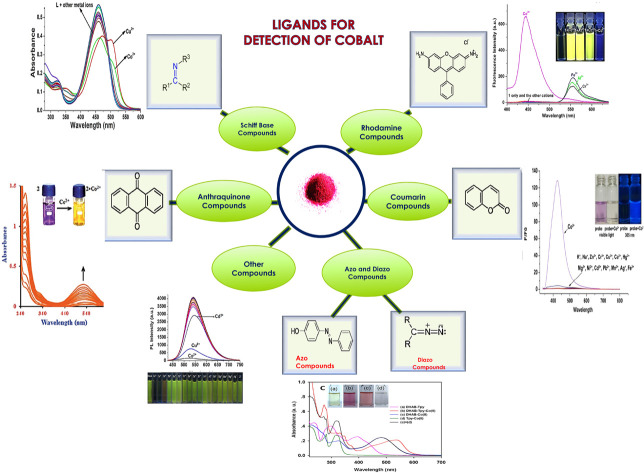

## 1. Introduction

Due to anthropogenic activities, the environment is degrading sharply with the accumulation of organic, inorganic and organometallic species. Metals are biologically non-degradable and accumulate in various vital organs. Even their injection or exposure at a low concentration can cause fatal effects to human beings. Hence, the detection and removal of these pollutants from the environment have attracted significant attention from researchers. Various techniques have been employed to resolve this issue in the recent past and numerous chemosensors have been developed, among which research on the fluorimetric and colorimetric techniques has gained significant interest.

Heavy metal ions (atomic density greater than 5 g cm^−1^) are very toxic given that they cause significant oxidative damage to living organisms; however, their capability to tune the structural changes of chemosensors upon binding serves as a tool for their detection. A chemosensor is a chemical compound with abiotic origin that attaches to the analyte reversibly in result of electronic changes. It has three components, *i.e.* a receptor unit that binds with the selective analyte, a spacer, which helps in modifying the geometry of the molecule, and a photoactive unit, showing different properties upon binding of the analyte with the receptor unit. Generally, chemosensors are divided into three major categories as follows: (i) colorimetric sensors whose electronic properties are changed due to intra-molecular charge transfer (ICT),^1^ (ii) fluorogenic sensors related to photo-inducted electron transfer (PET),^2^ fluorescence resonance energy transfer (FRET),^3^ excited-state intramolecular proton transfer (ESIPT) and excimer-exciplex formation and (iii) electrochemical sensors (whose properties change due to redox reactions). F. Goppelsroder was the first person to utilize morin chelate to determine the aluminium ion (Al^3+^) in 1867.^4^ Admittedly, an upsurge in the detection of charged and natural species by chemosensors has been witnessed in recent years given that these species play a significant role in the environmental, medicinal and biological fields.^5^ Numerous fluorescent and colorimetric chemosensors have been synthesized for the identification of heavy metal ions but the development of cheap and ecofriendly molecules remains a challenging task for researchers. These probes with distinct responses to various metal ions offer substantial benefits for *in vivo* and *in vitro* investigation by providing rapid detection, high selectivity and sensitivity, ease of operation, and biological compatibility. Fluorimetric and colorimetric dual-detection assessments can not only provide a highly sensitive fluorescence evaluation of metal ions but also help in easy and inexpensive ‘naked eye’ detection of metal ions, and thus they are presently considered promising candidates for the detection of heavy metal ions including cobalt ions. These ions not only play a vital role in many biological processes but also proven to be lethal if exceeding their permissible limit.

Cobalt ions are mostly found in minerals, soils and rocks and their concentration in wastewater is up to 100 μg L^−1^. The overall presence of Co^2+^ in the human body falls between 1.1 and 1.5 mg, with 43% present in muscles/soft tissues and 14% in bone.^6^ Cobalt is the key component of vitamin B-12 and necessary for many biological processes such as fat metabolism, amino acids, hematopoiesis, erythrocytes, myelin, co-enzymes,^7^ DNA formation and production of red blood cells (RBCs).

Industrially, Co^2+^ ions are used in the generation of O_2_/H_2_ electrodes, destruction of impurities through sulphate radicals, and improvement of the electrochromic characteristics of tungsten oxide nanowires.^8^ Cobalt is used in jewellery, tyres, cosmetics, production of corrosion-resistant alloys, magnets, magnetic recording media, beer and furniture.^9^

However, although cobalt is one of the essential heavy metals, concentrations exceeding its permissible limit have adverse effects on human health. The presence of cobalt ions in the environment is the result of anthropogenic activities. The introduction of cobalt ions in the human body in large amount leads to a series of diseases such as skin allergies, skeletal defects, diarrhea, genetic mutations, dry cough, interstitial pneumonia, sputum, hypotension, pulmonary edema, hard metal disease, asthma,^10^ lung and heart diseases, vasodilation, decreased cardiac enlargement, dermatitis,^11^ Wilson's disease and Alzheimer's disease.^12^ A very high dose of cobalt ions is responsible for “beer drinkers cardiomyopathy”.^13^ Hence, the WHO has included cobalt metal ions in the list of carcinogens and categorized it in class 2B carcinogens.^14^

Different techniques have been used to detect cobalt ions such as voltammetry, UV-vis spectroscopy, atomic absorption spectroscopy (AAS), fluorescence, electrochemical method, high-performance liquid chromatography (HPLC), pH electrode method, surface-enhanced Raman scattering, thermometric titration method, light-emitting-diode (LED)-based photometry, electro-thermal atomic absorption spectrometry (ETAAS), fiber optic-linear array detection spectrophotometry, surface plasmon resonance (SPR), chemiluminescence, capillary electrophoresis, fast neutron activation analysis (FNAA), magnetic resonance imaging (MRI) and flow-injection analysis.^15^

Electrochemical and optical sensors are highly selective and sensitive for the detection of heavy metal ions by showing an optical signal due to the interaction of the analyte with their recognition element. Optical sensors are based on Raman spectroscopy, chemiluminescence spectroscopy, fluorescence spectroscopy, colorimetric spectroscopy, infrared spectroscopy and refractive index spectroscopy. Alternatively, electrochemical sensors detect metal ions in the form of an electrical signal depending on concentration. 3D printing and photolithography technology have led to the development of microelectrodes and fork-finger electrodes for the detection of metals. The present portable ion detection systems are a combination of materials based on traditional detection and novel devices, which have the advantages of simplicity, low cost, miniaturization and efficiency.^16–19^

Fluorescence spectrometry is very useful given that it is very easy, non-invasive and has great sensitivity (parts per billion/trillion). High selectivity and spatial and temporal resolution with real-time monitoring ability demand the synthesis of fluorescent probes for the detection of Co^2+^ ions.^20^ Hence, numerous approaches based on fluorescence have been applied for the analysis of cobalt ions with the assistance of quantum dots (QDs)^9^ and colourimetry detection of the metal target ions accurately with simplicity, specificity and low cost. With time, various colourimetric chemosensors for the detection of cobalt ions have been developed such as amidine, fluoran, dansyl-styrylquinoline, terpyridine-based iron, rhodamine, dithizone, Schiff base, coumarin, and quinolone-based cobalt sensors. Colourimetric sensors for the detection of cobalt ions are ideal for getting quantitative information through internal calibration of dual absorption with a change in colour, while fluorescence-supported chemosensors for the identification of metal ions have received significant attention for detecting metal ions in real samples and bio-imaging of target analytes in living cells.^6^

The detection of metal ions by a ligand involves the coordination between the ligand and metal ions. These interactions result in either an increase or decrease in the fluorescence intensity and sensors can be categorized as turn-on or turn-off sensors.^21^ The underlying principle for turn-on sensors is chelation enhancement fluorescence (CHEF), which explains the increase in fluorescence intensity in the presence of metal ions. Similarly, the behaviour of turn-off sensors, whose intensity is reduced upon interaction with metal ions, can be described by the chelation enhancement quenching effect (CHEQ). There are many underlying mechanisms, as listed below, which reduce or enhance the fluorescence intensity.^22^ Turn-on chemosensors are weakly fluorescent in the absence of metal ions due to the PET effect, which is based on the transfer of electrons between the excited state and ground state of a molecule. In the ligand, the lone pair located on the heteroatoms possesses a higher energy than the HOMO of the fluorophore. When the electron jumps from the HOMO to LUMO, the electron in the lone pair moves down to HOMO and prevents the return of the electron from the LUMO. However, in the presence of metal ions, the energy of the lone pair decreases compared to the HOMO, which prevents the PET process and promotes CHEF.^23^ Intramolecular charge transfer is the movement of charge between different parts of a molecule and results in the energy relaxation of molecules in the excited state. A molecule contains donor and acceptor parts, which move the negative charges or electrons located on the lone pairs of heteroatoms. These charges may already be present in the molecule or induced by the interaction of the ligand with metal ions. The polarity difference between the donor and acceptor moieties together with environmental factors such as nature of the solvent determine the strength of the ICT mechanism. The ICT effect is responsible for the change in the position of the emission band *via* a bathochromic and hypsochromic shift. A rearrangement in the structure of the ligand results in planar intramolecular charge transfer (PICT) and twisted intramolecular charge transfer (TICT).^24^ Certain fluorescent compounds show high fluorescence properties in the aggregated form compared to solution form through a process called aggregation-induced emission. This phenomenon is the opposite to the aggregation-caused quenching (ACQ) mechanism. AIE was first introduced by Tang *et al.* to counter the issues resulting from ACQ in 2001. This gave an opportunity for researchers to develop a variety of chemosensors with biological applications.^25^ Fluorescence resonance energy transfer is a phenomenon in which energy transfer occurs between the donor and acceptor moieties. Intermolecular dipole–dipole coupling causes the non-radioactive energy transfer between the distant parts of a molecule and transfer is even more effective if the donor and acceptor parts of the molecule are located inside the Förster radius. The interaction of the ligand with the metal affects the amount of FRET process, which consequently changes the emission intensity.^26^ Excited-state intramolecular proton transfer (ESIPT) is the process in which intramolecular proton transfer occurs between the donor and acceptor moieties. However, these molecules undergo ESIPT given that they have increased acidic or basic nature due to the polarity difference between their donor and acceptor parts. For example, the electronegativity difference between the hydroxyl and carboxylic groups in salicylic acid causes a proton shift between them, resulting in the formation of zwitterions. Generally, these compounds show two emission peaks due to the presence of enol and keto forms.^27^ When a metal interacts with the ligand, it induces red or blue shifts, as well as hypochromic or hyperchromic effects, which are responsible for the detection of this metal ion. Metals ions have the ability to change the position of the emission band of the ligand in the emission spectrum. If the emission band shifts to a shorter wavelength, then it results a blue shift or hypsochromic shift. Similarly, sometimes the metal shows the opposite effect on the wavelength and shifts the band to a longer wavelength, which is referred to as a red shift or bathochromic shift. Moreover, an increase in the fluorescence intensity is a hyperchromic effect, while a reduction in intensity is referred to as a hypochromic effect.^28^

**Fig. 1:**
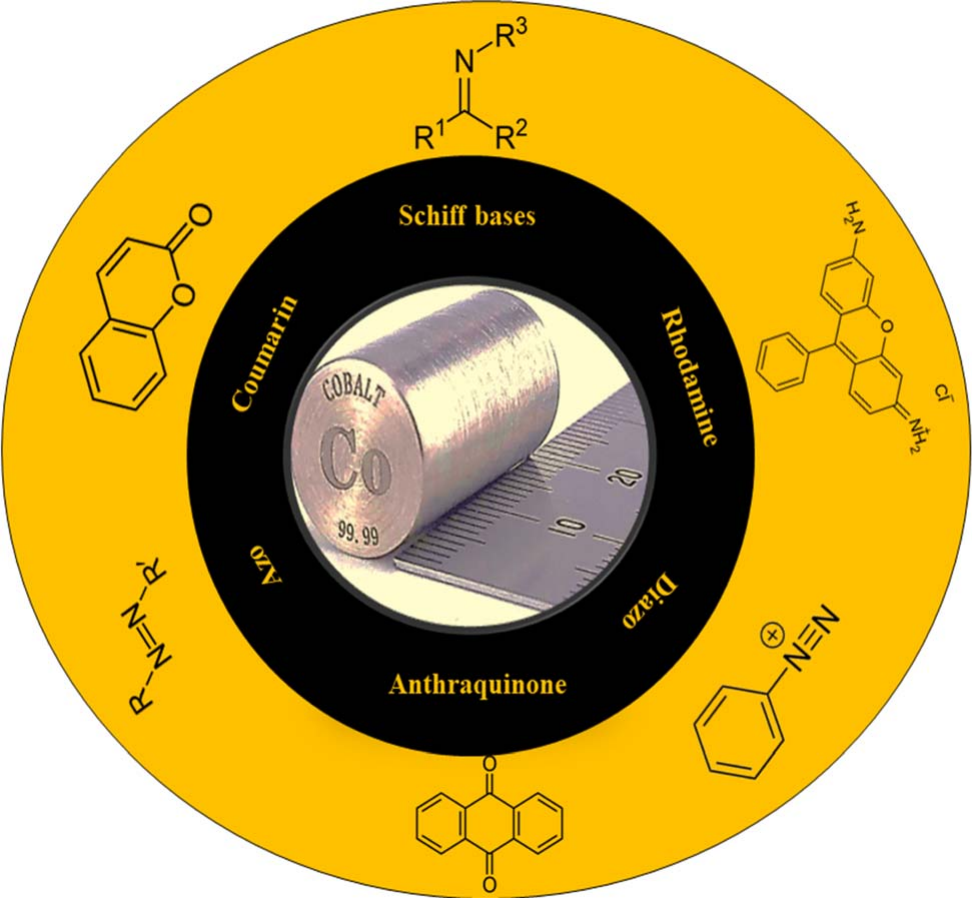
Different ligands for the detection of cobalt ions.

However, although chemosensors provide a simple tool for the detection of metal ions, they still have some challenges that need to be addressed. Improved selectivity is a great challenge given that many metals possess similar properties, which increase the chances of interference in the detection process. This can be avoided in a controlled system but in complex biological systems, a variety of metals or biomolecules is present, and thus interruption is very common. Interference can lead to completely opposite results. Moreover, interference is not only limited to other metals, where the behavior of chemosensors also depends on the surrounding environment such as the solvent and matrix interference. Similarly, chemosensors work at a specific pH and its variation affects both the selectivity and sensitivity. A variation in pH in complex systems reduces the liability and accuracy of the results. Also, is not possible to change the pH of biological systems as desired, which limits the use of most chemosensors in living systems. Despite all these limitations, chemosensors provide an easy and reliable tool for the detection of metal ions in a controlled environment. In this review, various ligands for the detection of cobalt ions are categorized into certain classes such as Schiff bases, rhodamine compounds, anthraquinone compounds, azo and diazo compounds, coumarin compounds and others compounds (Fig. 1) with specific emphasis on their use as fluorimetric and colorimetric chemosensors as well as computational study. Furthermore, the latest research in the field of nanomaterials for the detection of cobalt(ii) ions is also highlighted. This review will give inspiration on the development of more sensitive and specific chemosensors for cobalt in various types of samples.

## 2. Ligands for the detection of cobalt ions

Various types of ligands have been synthesized *via* the ultra-sonication, mechanical stirring, micro-wave-assisted and reflux techniques, which are very easy, quick and cost effective. However, the search for greener approaches is still in progress. In this section, different ligands are classified into different categories.

### 2.1. Schiff bases

Schiff bases are compounds having C=N bonds, which are formed from the condensation between an amine and carbonyl compound, and have been extensively employed as chemosensors for the detection of metal ions. Schiff bases, which have pi (π) electrons in their C=N group, are easy to synthesize and show good chelation with transition metal ions. Additionally, Schiff base derivatives having a chromophore and fluorophore are very useful tools for the visual sensing of metal ions. Schiff bases having π electrons and a nitro group from an aromatic ring exhibit good chelation by increasing the LMCT (ligand to metal charge transfer) and ICT (intramolecular charge transfer) transitions, which help in the detection of metal ions.

**Fig. 2:**
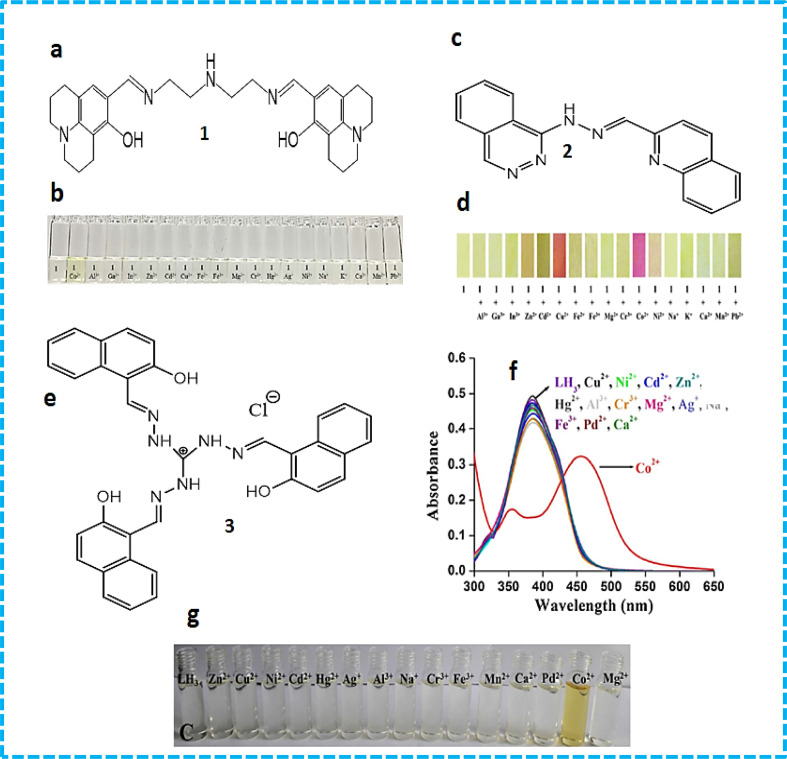
(a) Structure of sensor 1. (b) Colour changes in sensor 1 in the presence of metal ions. (c) Structure of sensor 2. (d) Colour changes in sensor 3 in the presence of metal ions. (e) Structure of sensor 3. (f) Absorption spectral changes with different metal ions. (g) Colour change with cobalt ions.^30^

Seong Youl Lee *et al.* synthesized dual chemosensor 1, which has a Schiff base together with a julolidine moiety, showing good complexation with transition metals. In addition, chemosensors having two binding sites can link with metals tightly, which gave the idea of the formation chemosensors that have two imino-julolidine groups. The linker used was diethylenetriamine. Dual chemosensor 1 in air turned a solution of cobalt ions from colourless to yellow (Fig. 2b). Under aerobic conditions, when Co^2+^ binds to receptor 1, it changes its oxidation state to Co^3+^. With an increase in the concentration of cobalt ions, distinct spectral changes were observed at 450 nm, with a decrease in intensity and two isosbestic points at 350 nm and 417 nm. Chemosensor 1 was subjected to density-functional theory (DFT) calculations to determine the potential binding site for the incoming metal ions. The hydrogen bond that exists between the OH and imine nitrogen groups stabilizes the sensor and it possesses a bent structure having bond lengths of 2.5963 Å and 2.5941 Å. The HOMO–LUMO transitions helped in the determination of the first lowest excited state, which revealed that intermolecular charge transfer occurs from julolidine to the imine group. The lowest transition state (fourth) was related to the visible region transitions, which were labelled as the LMCT band, causing a yellow colour to appear. This LMCT showed changes in the molecular orbital (MO) from the imine and julolidine groups to the metal-centered orbitals. Chemosensor 1 also showed a great enhancement in fluorescence intensity at 450 nm when Zn^2+^ was added to it, which revealed that sensor 1 can be an efficient tool for the fluorometric detection of zinc metal ions and colorimetric detection of cobalt metal ions.^29^

A novel Schiff base chemosensor 2 was prepared by Jae Jun Lee and colleagues by linking quinoline-2-carboxaldehyde with 1-hydrazinophthala-zinc in methanol using both quinoline and phthalazine groups, which was characterized by electrospray ionisation mass spectrometry (ESI-MS), ^1^H NMR, and elemental analysis. Receptor 2 showed significant sensitivity towards Cu^2+^ ions (yellow) and Co^2+^ ions (pink). Both Cu^2+^ and Co^2+^ were detected simultaneously using a colourimetric test kit coated with 2. Co^2+^ and Cu^2+^ showed peaks at 512 nm and 472 nm, respectively. The addition of Co^2+^ ions resulted in a decrease in absorption at 382 nm, while a new sharp band at 512 nm appeared. The formation of a complex and the colour change of sensor 2 was observed to be due to LMCT and ICT. The LMCT showed the molecular orbital changes from –NH– and –NvN– of hydrazinophthalazine to metal-centered orbitals, while the changes in the ICT were similar to that of 2. In the case of the Cu^2+^–2·1 complex, the excited states (492.02 and 483.05 nm) were found to be due to the colour change (orange) showing ICT, d–d transition and a bit of LMCT.^31^ A novel probe 3 C_3_-symmetric tri-aminoguanidine-2-naphthol conjugate was synthesized from a simple precursor, which selectively sensed Co^2+^ ions by a colourimetric test kit. Upon the addition of Co^2+^ ions, the colour changes were easily noticed by the naked eye. The addition of cobalt ions shifted the absorption band from 390 nm to 457 and colour of the solution changed from colourless to yellow (Fig. 2e and f). The molecular structure of 3 contain heteroatoms and bonds, which facilitate the rotation and easy transfer of charge in the molecules. Its emission was quite weak in DMF solvent due to its frequent isomerization and molecular rotation. However, during aggregation, both processes were inhibited, which enhanced its emission. Sensor 3 showed a great response in the determination of cobalt metal ions in tap water and drinking water.^30^

**Fig. 3:**
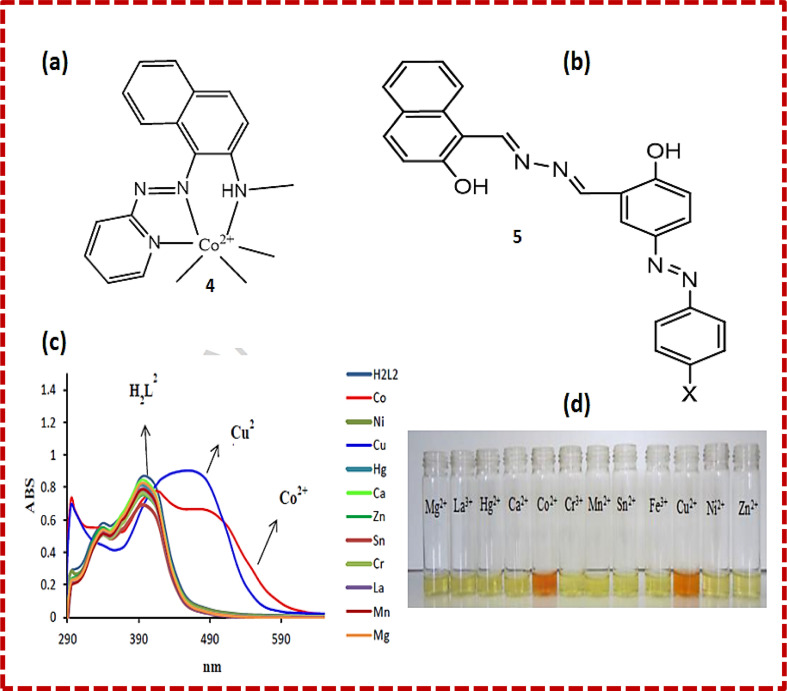
(a) Structure of 4-Co^2+^ complex. (b) Structure of sensor 5. (c) UV-vis spectra and (d) colour changes upon the addition of different metal ions to 5.^33^

Xue Bai *et al.* synthesized sensor 4 (Fig. 3a) based on the conjugation of heavy metals with a 1-(2-pyridylazo)-2-naphthol derivative in aqueous medium, which was characterized by energy-dispersive X-ray fluorescence spectrometry, FTIR (Fourier transform infrared spectrometry) and SEM (scanning electron microscopy). Calorimetric sensor 4 showed great selectivity toward Cu^2+^ and Co^2+^ ions and was used to determine Cu^2+^ and Co^2+^ in wastewater. Sensor 4 exhibited UV-vis (ultraviolet-visible) absorption at 480 nm. On the addition of Co^2+^ ions, two peaks at 450 nm and 575 nm were observed. Sensor 4 showed this response due to the high thermodynamic affinity of Co^2+^ and Cu^2+^ for rapid metal-to-ligand binding kinetics and N-donor ligands. The efficiency of the sensor was tested in real water samples. The colour of the solution changed from orange to brown for cobalt metal ions and purple for copper metal ions in water.^32^ Samira Gholizadeh Dogaheh *et al.* synthesized two new azo-azomethine receptors 5 (Fig. 3b) containing naphthalene, hydrazine and electron-withdrawing groups (NO_2_ and Cl), which was characterized by UV-vis spectroscopy, FT-IR, MALDI-TOF (matrix-assisted laser desorption/ionization-time of flight) mass analysis and elemental analysis. Its sensing ability towards various metal cations (chloride salts of Cu^2+^, Hg^2+^, Ca^2+^, Co^2+^, La^3+^, Ni^2+^, Mg^2+^, Zn^2+^, Sn^2+^, Cr^3+^ and Mn^2+^) was investigated based on its absorbance. The Cl group in chemosensor 5 was responsible for the detection of copper ions. Alternatively, the NO_2_ group in the sensor recognized both copper and cobalt ions. The absorbance of the chemosensor solution shifted from 400 nm to 450 nm (Fig. 3c) with a colour change (yellow to orange), which was attributed to the charge transfer (CT) (Fig. 3d). The binding ratio was found to be 1 : 1.^33^

Another Schiff-based chemosensor 6 was synthesized for the detection of cobalt ions. The colour of the solution of chemosensor 6 changed from pale-yellow to red when interacting with a solution of Co^2+^ ions. The UV-visible spectrum of the ligand showed a red shift from 368 nm to 480 nm with 25% increase in the absorption intensity of the ligand solution. The sensor also exhibited a response to zinc and copper ions but it was smaller than that for cobalt ions. Job's plot analysis confirmed the formation of a 1 : 1 complex. The intramolecular charge transfer (ICT) mechanism explained the interaction between Co^2+^ ions and chemosensor. The fluorescence spectrum of chemosensor 6 showed an emission band at 428 nm, which changed to 400 nm in the presence of cobalt ions. The chelation-induced fluorescence quenching was mostly induced by the redox-active Cu^2+^ and Co^2+^ ions due to the ligand-to-metal electron or energy transfer mechanism.^34^

Fasil and co-workers reported the preparation of chemosensors 7^1^ and 7^2^, which showed sensitivity towards nickel and cobalt ions, respectively. Fluorescence studies were carried out in DMSO–H_2_O at pH 7.5. Chemosensors 7^1^ and 7^2^ were synthesized to bind metal ions through the enamine N groups and carbonyl O as donors. The hydrogen bonding network was the main factor directing the thiazole ring, which is rotated 18.75° from the carbonyl group. The sulfur atom, cation binding pocket and carbonyl group were all pointing in the same direction. However, given that nitrogen is a much harder base compared to sulfur, the rotation occurred about the C21–C22 bond. This made a pocket of hard donors, *i.e.*, the carbonyl oxygen and imine nitrogen, which are better ligands for Ni^2+^ and Co^2+^ ions. The UV-visible spectra of the probes contained small and weak absorption peaks above 400 nm due to their open-ring structure. Cobalt and nickel ions caused a red shift in the absorption peak of the probe and a new band appeared at 500 nm with an enhanced absorption intensity. The bathochromic shift caused the changed probe's solution to yellow. The probe produced a fluorescence emission when excited at 470 nm. Cobalt ions shifted the emission by 15 nm towards a shorter wavelength to produce an emission peak at 515 nm. Cobalt ions also enhanced the fluorescence intensity of the probe. Nickel ions changed the colour of the probe, making the probe selective for them. These metal ions caused the opening of the spirolactam form. The oxygen of the carbonyl and nitrogen of enamine groups helped in the binding of the chemosensors with the metal ions. A 1 : 1 stoichiometric complex was produced upon complexation. The stoichiometric complex ratio was confirmed by the Job plot analysis. Ethylenediaminetetraacetic acid (EDTA) reversed the effect by removing the metal ions and regenerating the closed spirolactam form.^35^ Chemosensor 8 was synthesized by Smita and co-workers through the condensation reaction between 1-(pthalazine-4-yl) hydrazine and 2-acetyl pyridine. It was characterized by single X-ray crystallography and other sophisticated analytical tools. The absorption spectra of probe 8 showed two absorption peaks at 283 nm and 420 nm. Cobalt ions shifted the absorption peak at 420 nm to 470 nm. The peak intensity deceased due to the addition of cobalt ions. It changed the yellow-coloured solution of the chemo sensor to red. The shift in the absorption wavelength was observed due to the intermolecular charge transfer between the different groups of the chemosensor. The fluorescence study of the chemosensor exhibited an emission peak at 478 nm, which shifted to 463 nm after the addition of Co^2+^ ions with prominent quenching. Hydrogen bonding of the –NH group is a major feature of host–guest complexation. The delocalized amine hydrogen atom forms an intramolecular hydrogen bond with the nitrogen atom of the phthalazin and azomethine groups. This interaction is termed amine-hydrogen-to-imine-nitrogen separation. Sensor 8 showed intramolecular H-bonding, where the hydrogen atom of the amine group forms strong intramolecular H-bonds with N⋯N and C⋯N distances of 3.0468 and 3.620, respectively. The longer bond distance of sensor 8 showed solvent-assisted keto tautomerization, which led to a derivative of pyridine with enhanced chelation in the presence of Co^2+^, showing a normal fluorescence intensity due to an intramolecular charge-transfer (ICT) process. Copper, ferric and nickel ions interfered but their response was very small as compared to cobalt ions. Theoretical calculations were performed on isolated molecules, revealing differences in the bond parameters between the calculated and experimental values. The overlayed optimized structure of 8 with the X-ray crystal structure resulted in a root mean square error of 0.341, which confirmed the difference between the experimental and calculated values. The tautomeric form of 8 is 11.37 kcal mol^−1^ less stable than its normal form. Similarly, the interaction of the ligand–metal complex is 358.88 kcal mol^−1^ lower than that of the ligand, which indicates the stable complex formation. The frontier molecular orbital study showed charge transfer from the ligand to cobalt ions.^36^ Chemosensor 9 was developed *via* the ultrasonication method. A solution of probe 9 was prepared in ethanol. Different metal ions were added to this solution and their absorbance was checked. Absorption peaks were observed at 272 nm and 382 nm. After the inclusion of Co^2+^, the absorption was shifted to 679 nm, which showed the binding of the probe with the metal due to the d–d transition. The stoichiometry was found to be 1 : 1. The colour of the solution was observed to change to yellow from brown when the cobalt ion bound with the probe, which was explained by the chelation enhancement fluorescence (CHEF) mechanism. With the addition of different concentrations of cobalt, a gradual enhancement in fluorescence was observed. The absorbance intensity of the probe with cobalt ions was found to be the maximum at pH 7.3 and decreased from pH 8 to pH 14. The reversibility of the cobalt complex was checked by adding 1 μM EDTA. It was observed that the peak of the complex was quenched at 580 nm. The addition of cobalt metal ions to a solution of sensor 9 resulted in the formation of a four-coordinated complex of cobalt with the oxygen of the carbonyl group (C=O) and nitrogen of the imino group (C=N). Isomerization was restricted because of the C=N bond, which led to an enhancement in fluorescence and CHEF when Co^2+^ was added to the solution of sensor 9. Intramolecular charge transfer was observed from the N-atom to O-atom, which formed a chelate, resulting in an enhancement in fluorescence. Sensor 9 showed great response in terms of cytotoxicity and real water samples.^37^ Dipodal-based Schiff base chemosensor 10 was prepared by dissolving 2,6-diformylpyridine in methanol, and adding it to a solution of 4-fluorobenzylamine. The yield obtained was 90% and the structure of the crystal was found to be triclinic according to the crystallographic studies. A solution of cobalt nitrate (2.5 × 10^−3^ M) was mixed with a solution of (2.5 × 10^−3^ M) 10 in a 1 : 2 ratio. The colour of the solution of sensor 10 changed from light-pink to orange after the addition of Co^2+^ ions and the absorption band at 370 showed an increase in intensity. The charge transfer (CT) mechanism explained the complexation and the binding constant (log K) was found to be 4.5. Sensor 10 was found to be very useful for the detection of cobalt ions in aqueous medium.^38^ Aldehyde-based chemosensor 11 was designed using terephthaldehyde and p-amino benzoic acid. Nanoparticles of chemosensor 11 were prepared *via* the re-precipitation method, which showed an absorption band at 372 nm, while the emission was recorded at 427 nm and 461 nm. This was attributed to the aggregation-induced enhanced emission (AIEE). NPs-11 showed a longer fluorescence time than compound 11 in methanol due to the restriction of the molecular vibration and rotation during the formation of the nanoparticles. The fluorescence intensity of NPs-11 showed quenching at wavelengths of 427 nm and 461 nm. The absorption of NPs-11 was attributed to the head-to-tail arrangements of 11 nanoclusters by intermolecular π-stacking (J-aggregates). The LOD was found to be 0.102 μg mL^−1^ (1.73 μM) for cobalt ions. Chemosensor 11 showed a great inhibitory response towards Gram-positive (*Bacillus* sps.) and Gram-negative (*Escherichia coli*) bacteria, outstanding anti-TB response against *M. tuberculosis* H37RV and efficient detection of cobalt metal ions in real water samples.^39^ Schiff bases can strongly coordinate with many metal ions. If a Schiff base has a phenolic group, then it can target anions *via* hydrogen bonding. Hence, Schiff base sensor 12 was prepared from 1,8-hydroxyjulolidine-9-carboxaldehyde and 2-hydrazinyl-4-(trifluoromethyl) pyrimidine. A solution of probe 12 was treated with cobalt ions, which decreased the absorption intensity at 375 nm, while increasing the intensities at 265 nm and 427 nm. The transition in the 12-Co^+3^ complex was explained by ICT and LMCT. A slight pale colour was observed due to the binding of sensor 12 with Co^2+^. The detection limit was 0.18 μM. The pH range for the detection of cobalt was determined to be 6.0–10.0. The reversibility was checked by EDTA and the results confirmed that the colour of the solution remained the same by adding EDTA to the solution of complex. Density functional theory was applied to study the minimized energy structures of ligand 12 and the ligand–metal complex. Calculations showed that the orange color of the complex was due to the 16th lowest excited state and increased ICT transition. The theoretical results were consistent with the experimental values. Chemosensor 12 showed efficient detection of cobalt metal ions in real water samples.^40^ The preparation of pyrene derivative 13 was also reported. Sensor 13 appeared blue under a UV-lamp when cobalt ions were added with a binding ratio of 1 : 1. A good linear relationship between 13 and Co^2+^ was observed at 390 nm. The increase in the intensity of 13-Co^2+^ was due to chelation-enhanced fluorescence (CHEF). The fluorescence reversibility was checked by EDTA, which showed 90% recovery in the first three cycles. These results confirmed that sensor 13 could be reused nearly four times for the efficient detection of cobalt.^10^ Chemosensor 14 was prepared by the condensation of di-2-pyridyl ketone (14) with 6-hydroxypicolinohydrazide in ethanol for the detection of cobalt. Different metal cations were tested by mixing them with 14 in buffer (pH 7.0). The absorbance of sensor 14 at 314 nm decreased and there was an increase at 430 nm when a solution of cobalt ions was added. The absorbance was found to be the maximum at a 0.7 molar fraction of 14. The binding ratio was found to be 2 : 1. Due to the formation of the 14-Co^2+^ complex, the colour changed to yellow, which confirmed the complexation. ^1^HNMR titrations revealed that all the peaks disappeared with the addition of cobalt due to the paramagnetic property of cobalt. Hence, 14 could be used for the efficient detection of Co^2+^ in water samples.^41^ Chemosensor isatin-3-phenylhydrazone 15 was prepared by dissolving phenylhydrazine in a suspension of isatin in 10 mL of ethanol. The completion of the reaction was checked by TLC and the final product was obtained in the form of a yellow solid. A solution of chemosensor 15 was treated with different metal cations. Among them, only cobalt ions changed its colour yellow to orange. The complexation between the ligand and metal was due to charge transfer (CT) and a new band appeared at 520 nm. Chemosensor 15 showed effective results in the pH range of 6–3. Its LOD was determined to be 734 μM. The UV-visible spectral results showed that chemosensor 15 had better selectivity for Cu^2+^, Cr^3+^, and Co^2+^. Density functional theory (DFT) showed the formation of a 3D structure and the process of charge transfer occurred during the binding of sensor 15 with Co^2+^. The complexation of 15-Co^2+^ could be recovered due to the inclusion of EDTA with a colour change (orange to yellow). Furthermore, the qualitative recognition of cobalt was done by silica gel, in which significant colour changes were observed. Practical applications were performed by applying this ligand to water samples for the determination of cobalt. Chemosensor 15 showed excellent results in this regard.^42^

**Fig. 4:**
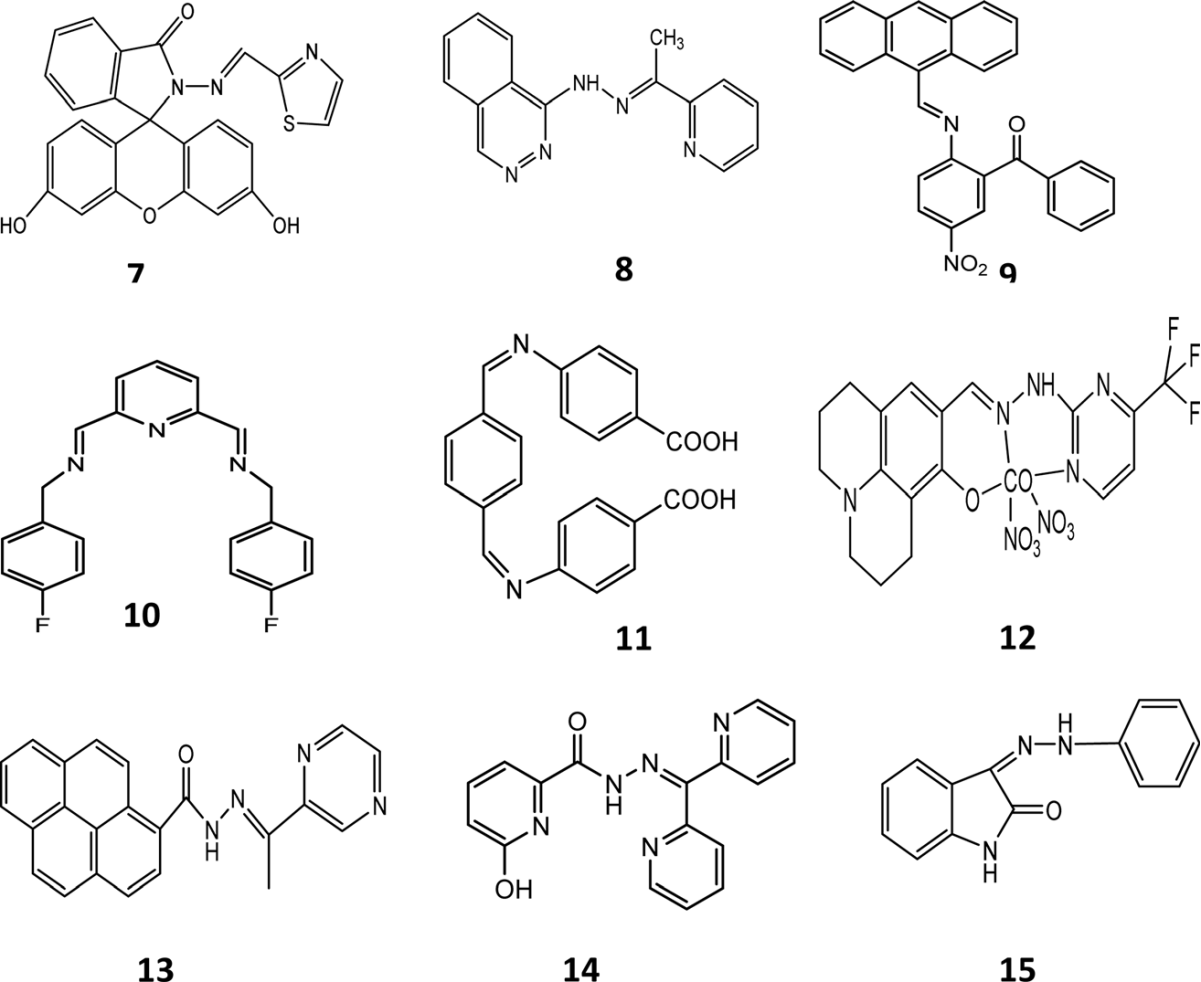
Chemical structures of chemosensors 7–15.

A camphor-derived fluorescent probe (16) was synthesized in ethanol for the detection of cobalt. A stock solution of 16 (5 × 10^−6^ M) was prepared in tetrahydrofuran (THF), while stock solutions of various metal ions were prepared (1 × 10^−4^ mol L^−1^) using their chloride salts and their emission recorded. The excitation wavelength was selected as 365 nm and the emission was recorded in the range of 500–700 nm. The presence of cobalt ions decreased the intensity at 547 nm with a colour change (scarlet to orange-yellow). The intensity of the emission changed under the influence of the concentration of cobalt ions. UV-vis spectra revealed that there were two absorption peaks at 520 nm and 490 nm, which were weakened on the addition of Co^2+^ ions. The binding ratio was found to be 1 : 2. The large energy gap between the highest occupied molecular orbital (HOMO) and lowest unoccupied molecular orbital (LUMO) caused a reduction in fluorescent intensity, confirming the mechanism of interaction between Co^2+^ and 16. The detection limit was recorded as 0.925 μM (Fig. 4).

**Fig. 5:**
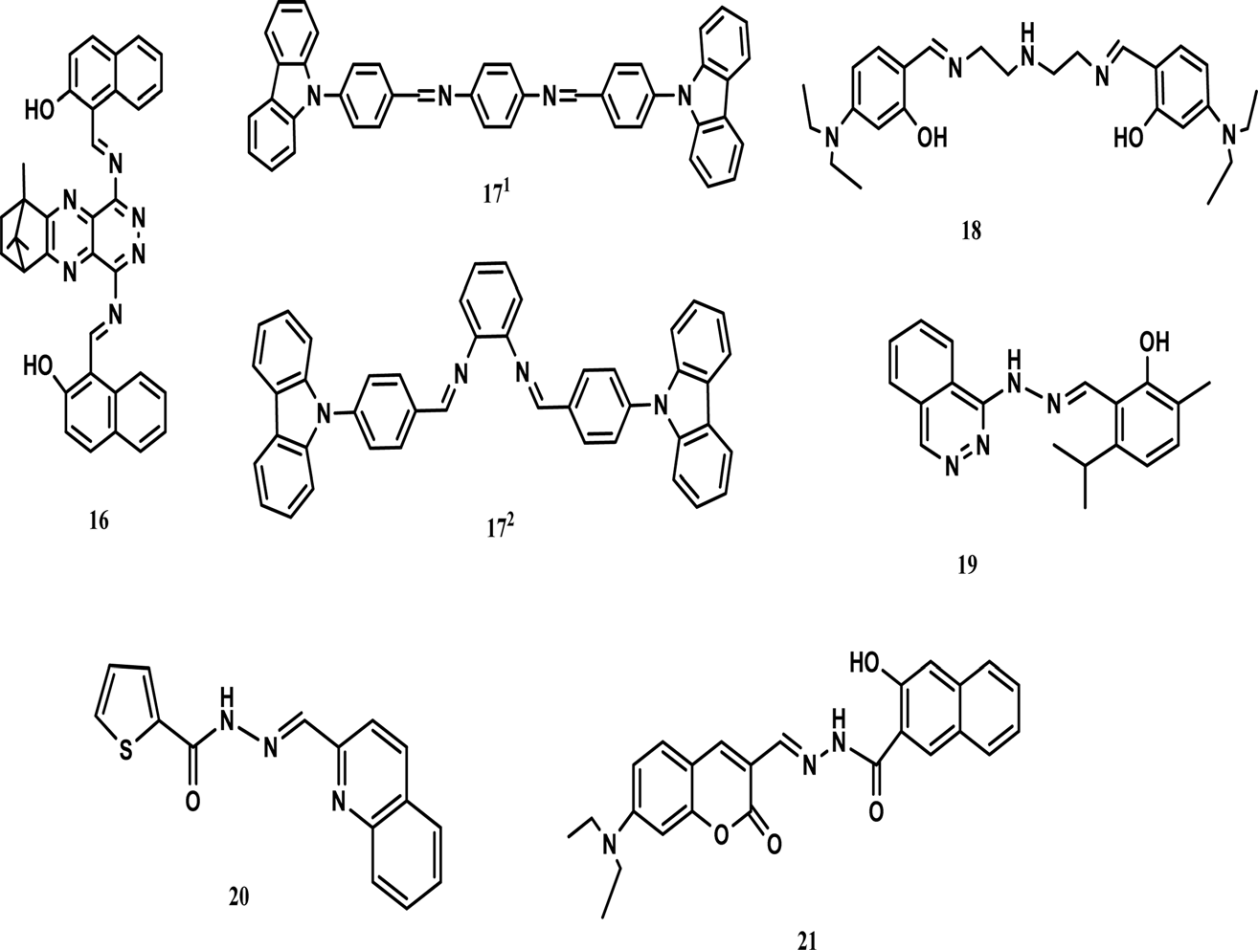
Chemical structures of chemosensors 16–21.

Density-functional theory (DFT) calculation was performed for the structural analysis of ligand 16 and the complex. The HOMO of 16 spread over the pyridazine group and the LUMO mobilized over the naphthalene group. This resulted in strong intermolecular charge transfer and prominent fluorescence. However, when a metal formed a complex with the ligand, then the HOMO accumulated on the naphthaldehyde group, while the LUMO dispersed over the other group. Fluorescence quenching occurred as the result of weak ICT. The energy gap between the HOMO and LUMO in the ligand was greater than that in the complex, which also stabilized the ligand.^14^ Juan and co-workers prepared two new Schiff bases, which were based on carbazole for the detection of cobalt and named 17^1^ and 17^2^. The optical properties of both probes were investigated using an F-4500 fluorescence spectrometer and UV-vis absorption. Compound 17^1^ showed absorption at 376 nm and emission at 495 nm, while compound 17^2^ showed absorption at 333 nm and emission at 397 nm. The emission spectrum of compound 17^1^ was found to be broad due to several allowed transitions, which occurred due to the poor conjugation on excitation. The fluorescence spectra of both compounds showed a red-shift when the polarity of the solvent was increased and 17^1^ showed a red-shift of 497 nm in DMSO. When 1.0 × 10^−8^ M of Co^2+^ was incorporated in the solution, the emission was enhanced due to the strong chelation-enhanced fluorescence effect (CHEF) of the ligand and cobalt. When cobalt was added to compound 17^1^, a blue shift occurred from 448 nm to 375 nm, while no shift occurred in compound 17^2^. This showed that compound 17^1^ was highly selective towards cobalt as compared to compound 17^2^. The detection limit was found to be less than 10^−14^ M.^43^ Chemosensor 18 was obtained by dissolving 4-diethylaminosalicylaldehyde and diethylenetriamine in absolute ethanol. A solution of sensor 18 was treated with cobalt ions, which resulted in a shifting in the absorption band at 360 nm towards 420 nm with change in the colour of the solution to yellow. The 18-Co^3+^ complex was optimized by diamagnetic character because the 18-Co^2+^ complex was oxidized to 18-Co^3+^. DFT calculations were performed to support the experimental data. A bent structure was found for the minimized structure with a dihedral angle of −142.971°. The HOMO–LUMO transition calculation showed that charge transfer occurred from the diethylaniline group to the imine group. The third lowest excited state of the complex showed predominant ligand to metal charge transfer (LMCT), which resulted in a colour change in the ligand. Sensor 18 showed the efficient detection of cobalt metal ions in real water samples.^44^ Smita and co-workers synthesized a unique chemosensor (19) *via* the condensation of 2-hydroxy-3-methyl-5-isopropylbenzaldehyde with 1-(pthalazine-4-yl) hydrazine in ethanol, which was characterized by the liquid chromatography-mass spectroscopy LC-MS, carbon-13 nuclear magnetic resonance (^13^C-NMR) and proton nuclear magnetic resonance (^1^H-NMR) spectroscopic techniques. A solution of receptor 19 was prepared in acetonitrile (CH_3_CN), while solutions of different cations were made in double-distilled water. Receptor 19 showed absorption at 286 nm and 383 nm. An obvious fluorescent enhancement was observed for cobalt compared to other metal cations due to the formation of a complex, *i.e.*, 19-Co^2+^. In the presence of cobalt ions, the absorption was shifted to 435 nm. Further, incremental doses of cobalt in a solution of 19 caused an increase in its absorbance. The ICT and CHEF mechanisms explained the binding. Three isosbestic points were observed at 291 nm, 325 nm, and 410 nm. An obvious colour change was observed. Due to the inclusion of cobalt, the yellow-coloured solution became green. The binding ratio was 1 : 1 and the detection limit was 25 nM. The reversibility of the receptor was checked by adding EDTA and the yellow colour of the receptor was recovered. Hence, this receptor could be used for the intracellular imaging of cobalt.^45^ A Chemosensor 20 based on thiophene-2-carbohydrazide was also prepared for the detection of cobalt (Fig. 5).

Changes in its absorption spectra and colour were recorded in the presence of metal ions. The peak at 325 nm shifted to 400 nm with a colour change (colourless to yellow), while no obvious change was observed regarding other metal ions. Cobalt ions caused the appearance of a peak at 400 nm with an isosbestic point at 356 nm. The mechanism of binding was supposed to be ligand-to-metal charge-transfer (LMCT) with a binding ratio of 2 to 1. The limit of detection (LOD) was calculated to be 0.19 μM and the reversibility was confirmed with ethylenediaminetetraacetic acid (EDTA). Chemosensor 20 showed its efficiency in the detection of cobalt ions in real water samples.^46^ A probe (21) was prepared by reaction of 3-hydroxy-2-naphthohydrazide with 7-diethylaminocoumarine-3-aldehyde in methanol. Probe 21 was obtained in the form of a yellowish-orange solid and its stock solution was prepared in dimethylformamide (DMF). Stock solutions of different metal ions were prepared in ethanol. These stock solutions were diluted with methanol–water (v/v = 7 : 13) for test sampling and their spectra were measured. At less than 450 nm excitation, probe 21 and fluorescein have the same emission region. Probe 21 showed an emission peak at 525 nm, which quenched the emission of the cobalt ions, while the addition of other metal ions had no prominent increase in intensity. The LOD was found to be 6.96 × 10^−8^ M for Co^2+^ ions. The binding stoichiometry was calculated to be 2 : 1. The phenomenon of static quenching was observed during complexation and the colour change (yellow to orange-red) was observed by the naked eye. These results proved that sensor 21 would be efficient for the detection of Co^2+^ in water samples and this probe could also be used for cell imaging.^47^ Another chemosensor was prepared from 8-hyfroxyjulodine-6-carboxaldehyde and quinulin-8-amine, which showed a colorimetric response toward cobalt(ii) ions, changing the colour of the solution from yellow to orange. The sensor worked best in the pH range of 4 to 6 in aqueous medium.^48^ Yang *et al.* synthesized a pyridyl-based chemosensor for the colorimetric detection of cobalt(ii) ions. The colourless solution of the chemosensor turned yellow in the presence of cobalt(ii) ions. The binding constant was calculated to be 2 × 10^9^ M^−2^ with the best operation under neutral conditions.^49^

The study of Schiff base chemosensors suggests that their commercial application is hindered by the maintenance of the pH of the system. Moreover, their limit of detection (LOD) is around the micro to nano level. However, they have non-toxic behaviour towards living cells, which enhances their biological importance, as confirmed by the MTT assay (3-(4,5-dimethylthiazol-2-yl)-2,5-diphenyl-2*H*-tetrazolium bromide). Living cells, *e.g.*, HeLa cells, incubated with Schiff bases remained alive even after a long time. Schiff bases show a significant change in their fluorescence properties when interacting with cobalt ions in real water samples. Another Schiff base chemosensor was reported, which showed colorimetric sensing ability. The colour of the sensor changed from pale yellow to brownish-orange when interacting with cobalt ions. The LOD was calculated to be 1.24 × 10^−7^ M and TD-DFT calculations supported the formation of a complex (Table 1).^50^

**Table 1:** Schiff bases for the detection of cobalt ions

Probe	Medium	Ex/Em (nm)	Association constant Ka/M	Limit of detection	Application	Ref.
1	Bis–tris buffer (pH 7)	373	1.1 × 10^4^	0.34 μM	Detection of cobalt in real water samples	29
2	Bis–tris buffer (pH 7)	512	1.0 × 10^10^	1.5 μM	Detection of cobalt in real water samples	31
3	DMF and water (pH 7.4, 8.2)	390/542	5.50 × 10^4^	135 nM	Detection of cobalt in real water samples	30
4	Sodium bisulfite and ammonium hydroxide	450	1.21 × 10^8^	0.22 μM	Detection of heavy metals in real water samples	32
5	THF (Tris–HCl buffer, pH 7)	340–400/450–550	4.580 × 10^6^	2.45 μM	Detection of cobalt in real water samples and HeLa cells	33
6	CH_3_OH/H_2_O	404/428	—	0.707 μM	Trials are in progress to use this ligand *in vivo* imaging	34
7	DMSO in aqueous solution	470/515	2.1 × 10^4^ (Co^2+^)	100 μM and 120 μM	Detection of cobalt in real water samples	35
			4.5 × 10^3^ (Ni^2+^)			
8	CH_3_OH/H_2_O	420/478	—	50 nM	Naked eye detection of cobalt ions in real sample	36
9	Ethanol	382	—	0.91 nM	Real water sample analysis	37
					Biomedical application	
					Cell imaging	
10	Water–methanol (v/v, 1 : 1)	370/570	4.5	—	Detection of cobalt in aqueous media	38
11	Methanol	372/427	—	1.73 μM	Detection of cobalt in real water samples, anti-TB activity, and anti-bacterial activity	39
12	THF (bis–tris buffer, pH 7)	265/427	1.0 × 10^4^	0.18 μM	Detection of cobalt in real water samples	40
13	DMSO–PBS buffer solution (6 : 4, v/v, pH 7.4)	345/370		0.104 μM	Detection of cobalt in real water samples and HeLa cells	10
14	0.01 M bis–tris, pH 7.0	314/430	3.0 × 10^9^	0.08 μM	Determination of cobalt in water samples	41
15	DMSO–water 1 : 1 (v/v)	520	2.85 × 10^3^	734 μM	Determination of cobalt in water samples	42
16	H_2_O–THF (v/v = 6/4, pH = 7.4)	365/547	—	0.925 μM	Determination of cobalt in water samples and vegetables	14
171 and 172	DMSO	495	—	Below 10^−14^ M	Determination of cobalt	43
		397				
18	Bis–tris buffer (pH 7)	420	—	0.65 mM	Detection of cobalt ions in real samples	44
19	CH_3_CN/H_2_O (1 : 1, v/v)	435	—	25 nM	Monitoring Co^2+^ in living cells	45
20	DMSO/bis–tris buffer solution	400	1.0 × 10^10^	0.19 mM	Determination of cobalt in water samples	46
21	Ethanol/water with phosphate buffer (pH 7.2)	*λ*_emi_ = 525 abs = 350 & 511	2.24 × 10^6^	4.55 nM	Determination of cobalt in water samples and cell imaging	47

### 2.2. Rhodamine compounds

Thiacalixarenes show many interesting features because of their bridging sulfur atoms. Various probes such as rhodamine, amino-anthraquinone, dansylchloride, naphthalene, and coumarin linked to thiacalixarenes are used for the detection of metal ions. Derivatives of rhodamine have excellent photophysical properties, such as high quantum yields, high extinction coefficients, great photostability, and long emission wavelengths. Derivatives of rhodamine spirolactone are colorless and nonfluorescent, while the ring-opening of spirolactam or lactone can result in strong fluorescence emission and appear pink in colour.

A fluorescent-probe TrisRh (22) was synthesized by adding tris(2-aminoethyl) amine dropwise to compound 2 in chloroform in a 1 : 1 ratio under the protection of nitrogen, forming a faint yellow powder. Sensor 22 was used as a turn-on fluorescent probe for Co^2+^ ions. Cobalt ions quenched the fluorescence at 510 nm to 700 nm and the colourless solution turned pink. The stoichiometry was verified by electrospray ionization mass spectrometry (ESI-MS). The binding model of the complex was deduced using the FT-IR and ^1^H-NMR spectroscopic methods. In the Fourier transform infrared spectra of sensor 22 and the 22–Co^2+^ complex, the specific peak of the amide carbonyl absorption peak in chemosensor 22 was switched from 1710 cm^−1^ to 1703 cm^−1^ upon the addition of Co^2+^ ions, confirming its coordination through the oxygen atoms of the amide carbonyl in sensor 22. The Benesi–Hildebrand equation was used to determine the association constant (6.68 × 10^5^ M^−1^).^51^ Another chemosensor 23 with a coumarin moiety showed high selectivity with a fluorescence turn-on response toward Co^2+^ ions and trivalent metal ions in methanol. It recognized Al^3+^ in aqueous medium, which has potential application to determine Al^3+^ in live cells. In spectrophotometric studies, the addition of Co^2+^ ions to a solution of sensor 23 resulted in the appearance of a peak at 532 nm and a colour change from yellow to red. According to the Job's plot experiment, it was inferred that the stoichiometry of the 23-Co^2+^ complex is 2 : 1. The selectivity of chemosensor 23 toward metal ions in H_2_O solution is important due to its AIRE property. According to the Benesi–Hildebrand equation, the binding constant was calculated to be 5.93 × 10^5^ M^−1^. Thus, chemosensor 23 has potential application for detecting Co^2+^ in live cells or H_2_O samples.^52^ Fluorescent sensor 24 synthesized from rhodamine-6G and pyrazine showed colorimetric and turn-on fluorescence towards paramagnetic Co^2+^ ions. It was characterized by ^1^H NMR, ^13^C NMR, and HR-MS. Treatment sensor 24 with Co^2+^ ions resulted in the appearance of an absorbance band at 527 nm with a colour change from colourless to pink-orange. Sensor 24 showed fluorescence at 550 when excited at 510 nm and exhibited a fluorescent turn-on response, which increased by 56-fold in the presence of Co^2+^ ions due to the ring-opening of spirolactam. The stoichiometry of the 24-Co^2+^ complex was calculated to be 2 : 1. This sensor can be used for the fluorescence determination of Co^2+^ in living cells and within biological samples. To understand the structural configuration of 26 and the metal–ligand complex, density-functional theory (DFT) calculation was performed. It showed that the ligand possesses a spirolactam ring, which was formed in the rhodamine molecule. In the complex, cobalt ions coordinated with 4 oxygen atoms and 2 nitrogen atoms from two ligands. It caused the restriction of the rotation the coumarin part in the complex, which significantly enhanced the fluorescence. The bond lengths remained normal. Half of the HOMO of 26 spread over the xanthene and hydrazone part of the group, while the LUMO was spread over the coumarin part of the molecule. In the complex, the HOMO was only spread over the xanthene group, and the LUMO over coumarin, which caused charge transfer from coumarin to rhodamine. The energy gap between the HOMO and LUMO for the ligand–cobalt complex was 2.72 eV.^53^ Biswonath and co-workers reported the synthesis of rhodamine derivative 25 by adding 2-chloromethyl pyridine hydrochloride and triethylamine in a stirring solution of a reduced Schiff base in acetonitrile. The UV-vis absorption spectrum of 25 in a THF–H_2_O system showed an absorbance at the wavelength of 272 nm and 315 nm. Probe 25 exhibited fluorescence quenching upon excitation at a wavelength of 500 nm and its solution turned pink. In the fluorescence studies, a peak at 582 nm appeared due to the formation of a complex. Hg(ii) ions also showed the same result in the fluorescent studies with a colour change but had a lower intensity compared to Co^2+^ ions. Both the chromogenic responses from colourless to pink and fluorogenic responses from non-fluorescent to fluorescent are because of the characteristic absorption and the fluorescence peaks can be attributed to the metal-ion-induced spiro-ring opening. The stoichiometry of complexation was 2 : 1 for the 25-Co(ii) complex and 1 : 1 for the 3-Hg(ii) complex. Sensor 25 can be used to identify Co^2+^ ions in plants and animals.^54^

**Fig. 6:**
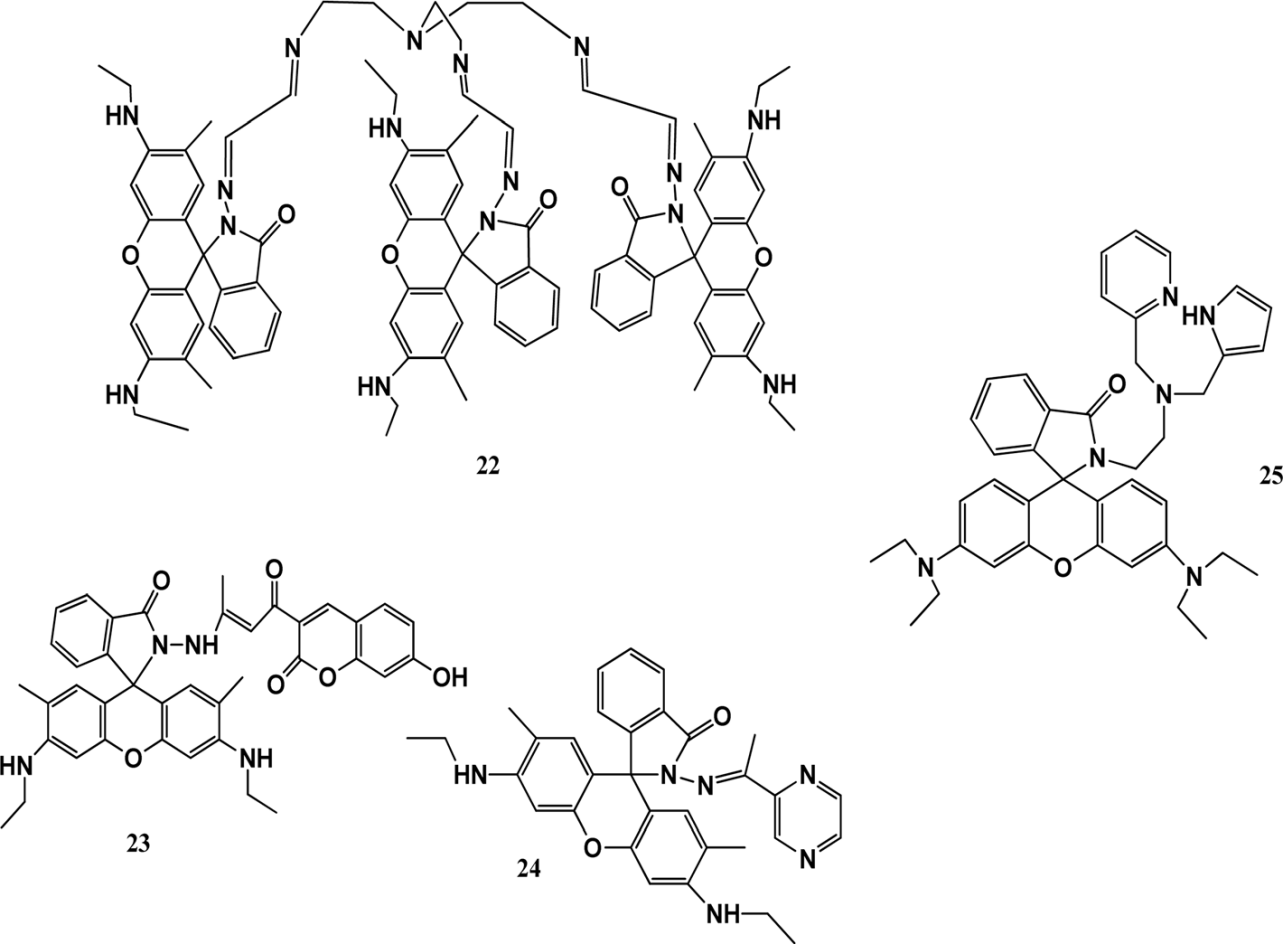
Chemical structures of chemosensors 22–25.

The study of rhodamine-based chemosensors has shown that they have unique structural properties and fluorescent abilities as their closed colorless spirocyclic structures change to the ring-opened form when they interact with cobalt metal ions, resulting in a prominent color change. These color changes can be easily observed by the naked eyes. Rhodamines have high absorption coefficients and fluorescent properties, and thus can be extensively used in different fields. Their unique structural features and fluorescent properties depending on the target metal ions make them good and affordable biomarkers and biosensors (Fig. 6).

They possess biocompatibility and can be used in living cells as biosensors without any damage. However, despite all the work reported on rhodamines as biomarkers and biosensors, there is still many gaps that need to be filled. Nevertheless, these sensors have a low limit of detection and can be made easily, and thus are promising candidates for bio-sensing at a commercial level.

Rhodamines contain two amino groups and one carboxylic group, which increase the structural possibilities to develop a variety of probes with different sensitivity and selectivity. Rhodamine-based chemosensors show spirolactam ring opening and closing in the presence and absence of metal ions, respectively, which improves and promotes their behavior towards the target ions with better sensitivity. Furthermore, their unique structural features and fluorescent properties, which depend on the target metal ions, make them good and affordable biomarkers and biosensors. Most commonly, these rhodamines interact with analytes through coordination bonds with heteroatoms especially nitrogen, oxygen and sulfur. However, some chemosensors detect metal ions through reversible reactions. Rhodamine-based chemosensors possess good solubility in water, which significantly enhances their applications in living organisms and environmental samples. Nevertheless, they possess good biocompatibility due to their high cell membrane permeability and low cytotoxicity, which make them good candidates for biological applications. However, although these rhodamine-based chemosensors show repeatability, accuracy and sensitivity, a lot of work is still needed to make them promising candidates for the detection of metal ions at the commercial level. Zhang *et al.* also reported the preparation of a rhodamine-based chemosensor for the effective detection of multi-metal ions. The colour of the sensor solution changed to pink for cobalt(ii) ions, and also exhibited reversibility on treatment with ethylenediaminetetraacetic acid (EDTA). The limit of detection (LOD) was reported to be 0.11 μM. The sensor was also applied in living cells and proved to be a potential candidate for target metal ions (Table 2).^55^

**Table 2:** Rhodamine compounds for the detection of cobalt ions

Probe	Medium	Ex/Em (nm)	Association constant Ka/M	Limit of detection	Application	Ref.
22	ACN/water	500/(525 in UV-visible and 544 in fluorescent titration)	6.68 × 10^5^	1.22 nM	Identification of cobalt ions in aqueous medium	51
23	H_2_O/CH_3_OH solution	385/445	—	—	Identification of cobalt ions in aqueous medium and live cells	52
24	THF/tris buffer solution (pH = 7.5)	510/550	—	0.31 μM	Detection of cobalt in human body with high selectivity and sensitivity	53
25	THF/HEPES buffer solution (pH 7.4)	500/582	—	4.3 nM	Detection of Co^2+^ ions in plants and human	54

### 2.3. Anthraquinone compounds

Anthraquinones have a high absorption coefficient, large Stokes shift, good photostability and maximum absorption or emission peaks located in the visible region, which develop a foundation for colorimetry. They are known for their redox nature and their change in fluorescent properties after interaction with metal ions and can be employed for redox sensing. Similarly, they can be linked with ionophores, which make them suitable for the detection of anions besides cations. Minor structural modifications in anthraquinone produce derivatives, which selectively detect anions or cations. Due to the inhibited intramolecular photoinduced electron transfer or excited state intramolecular proton transfer, these probes mostly show weak fluorescence, which are prominently enhanced after their interaction with metal ions. Thus, it can be concluded that these probes can be used for the detection of ions up to the ppm level and their employment in the colorimetric detection is very bright.

**Fig. 7:**
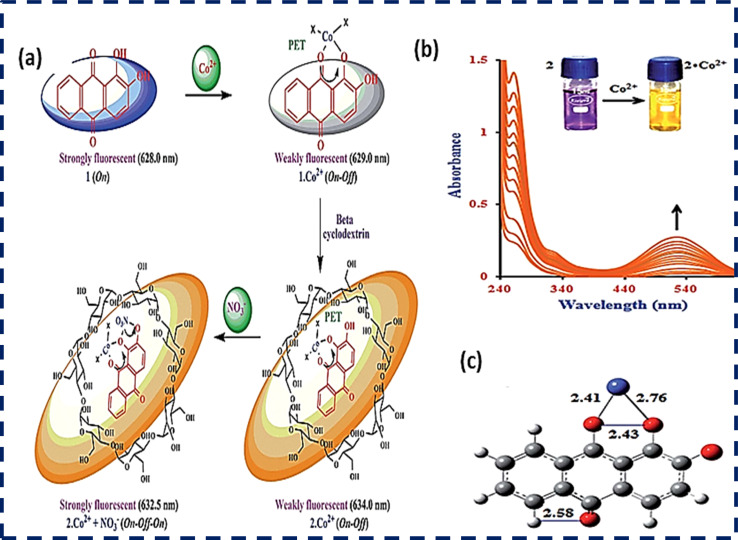
(a) Proposed sensing mechanism of Co^2+^ and NO^3−^ ions and (b) absorption spectrum of probe 28. (c) DFT-optimized structure of 28-Co^2+^. Ref. 57.

Qing-Xiang and co-workers synthesized probe 26 based on bis-benzimidazolium salt. A diffractometer was used to collect data for the chemosensor. The SHELXS program was used to determine the structures of chemosensor 26 and NCHN and phenolic hydroxyl signals were observed in the determination. A 5 × 10^−6^ mol L^−1^ solution of chemo sensor 26 was excited at 410 nm and a very broad emission peak appeared at 580 nm. This peak can be attributed to anthraquinone. When a 2.0 equivalent solution of cobalt ions was added, there was a sharp decrease in the fluorescence intensity at 580 nm and the solution turned red from orange. The capture of Co^2+^ ions by probe 26 caused the PET process to occur, which was responsible for the quenching of the fluorescence. The LOD was determined to 0.098 × 10^−6^ M for probe 26 and its detection limit was similar that reported in the literature, *i.e.*, 0.78 mM to 0.053 mM.^56^ Probe 27 was prepared and used for the detection of cobalt ions using fluorescence spectroscopy. The complex formed by the deprotonated phenolic oxygen of the probe with Co^2+^ ions shifted the peak from 530 nm to 522 nm (Fig. 7b). The calibration values were obtained between the maximum and minimum values of intensity and a straight cure was obtained for the graph. The limit of detection was found to be 22.7 nM using this approach. The biological applications of this chemo-sensor towards cobalt and nitrate ion complexes were proven using a human cervical cancer cell line. When these cells were observed using an excitation wavelength of 500 to 550 nm under a fluorescence microscope, they showed fluorescence emission in the range of 625 to 635 nm. These cells could take up the cobalt and nitrate ions and when complexes were formed with the probe, =they produced fluorescence (Fig. 7a). This indicates that when applied to biological systems, these receptors have the same fluorogenic properties. The optimized geometries of 29 and the complex were studied. *Ab initio* calculations showed that the catechol functional group of the ligand is the chelation site. The HOMO–LUMO excitation corresponding to the low energy excitation was related to the charge transfer between the catecholate and quinone molecules. However, calculations showed that the removal of cobalt ions could not restore the emission of ligand. The energy gap between the HOMO and LUMO was determined to be 1.16 eV.^57^

Studies have shown that anthraquinone possess exceptional optical properties and it has been extensively used as a chemosensor for the colorimetric detection of cobalt metal ions. Anion binding units such as urea whose acidity can be increased by attaching electron-withdrawing groups facilitate the rapid detection of cobalt ions. Obviously, an anthraquinone chemosensor showed great sensitivity when injected in the cancerous human cervical HeLa line cell, and also subjected to real field analysis of cobalt ions but a much work needs to be done for its commercial application (Table 3).

**Table 3:** Anthraquinone compounds for the detection of cobalt ions

Probe	Medium	Ex/Em (nm)	Association constant Ka/M	Limit of detection (μM)	Application	Ref.
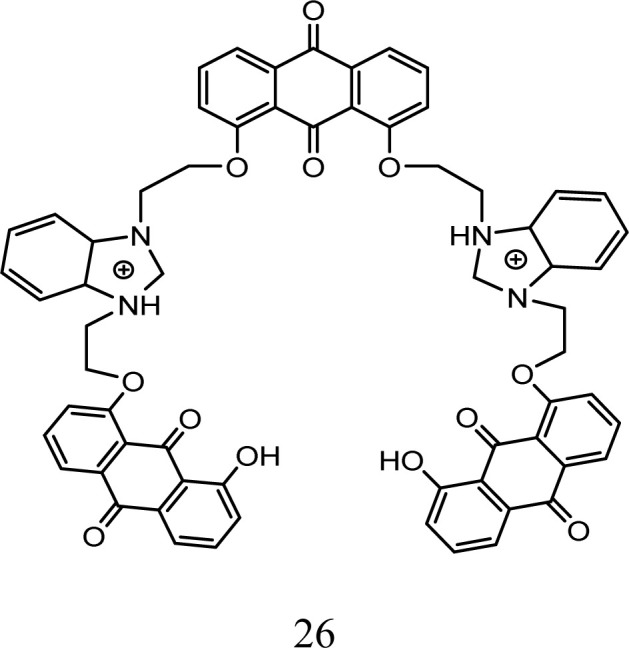	CH_3_CN/DMSO, THF, CH_2_Cl_2_	410/580	3.6 ×10^5^	0.098	Metal ion detection	56
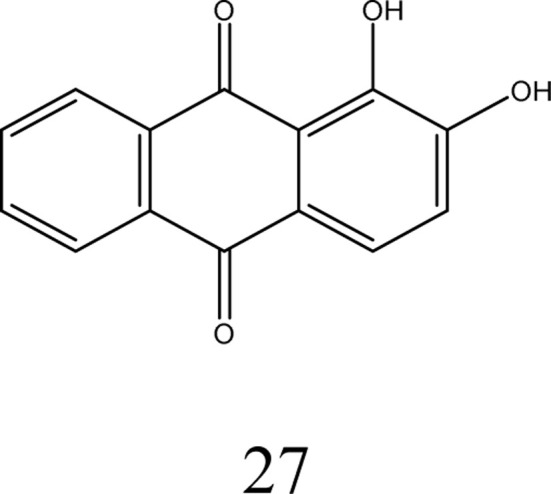	DMF/CDCl_3_/CD_3_CN, water	530/522	3.77 × 10^4^	22.7	Sensitive in cancerous HeLa line cells of human cervical	57

### 2.4. Azo and diazo compounds

Azo- and diazo-based chemosensors mostly reveal obvious color when interacting with metal ions and their absorption or emission maximum is located in the visible region, which make them a good choice for the naked-eye detection of metal ions. These chemosensors may also show *cis*–*trans* isomerization, which can be employed for the development of photo-responsive sensors. Due to the availability of functional groups, they provide flexibility in structural modification to develop a range of sensors.

**Fig. 8:**
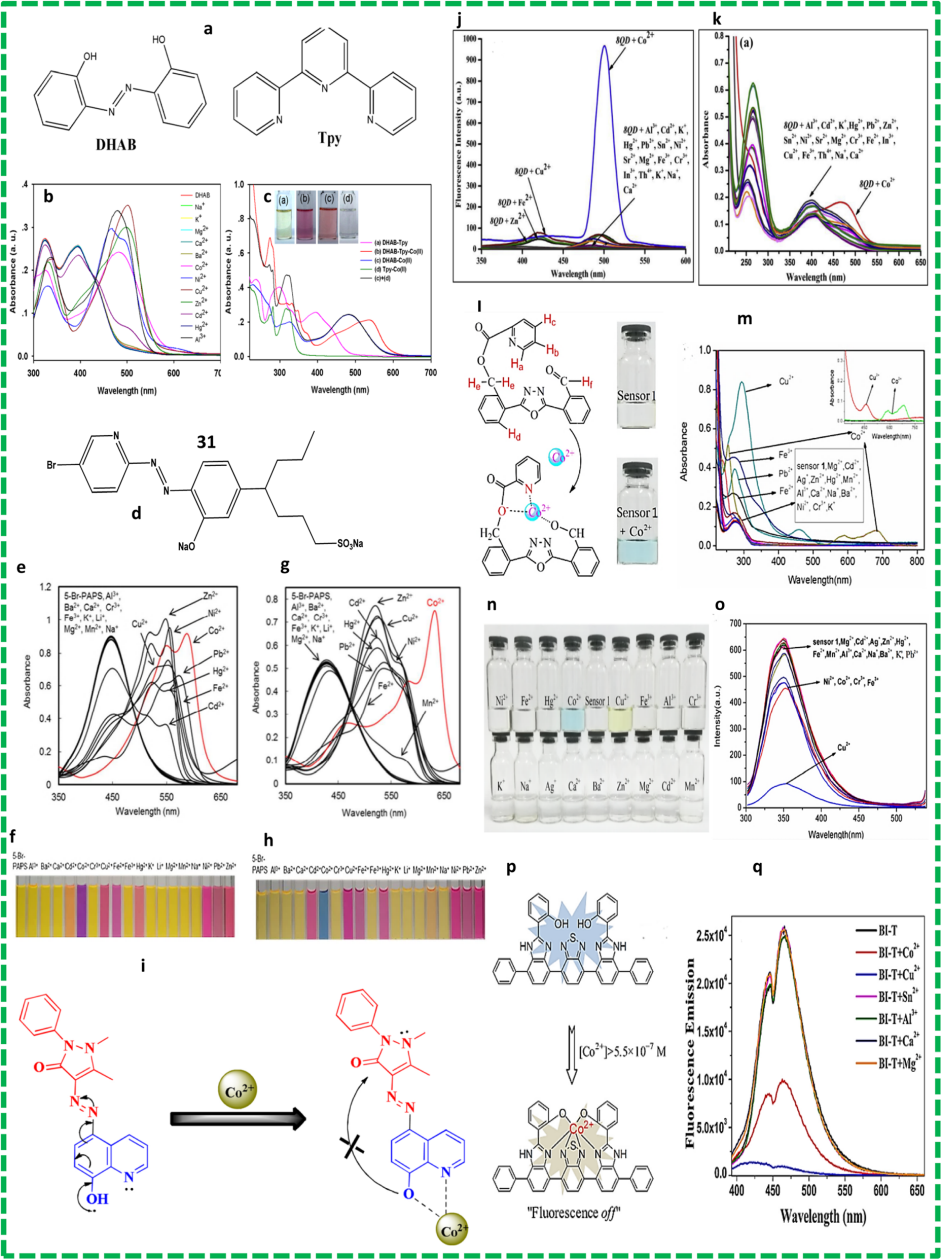
(a) Chemical structures of metal receptors 28 and (b) absorption spectra of DHAB (c) and Tpy with different metals.^58^ (d) Chemical structures of probe 29. (e) Absorption spectra of sensor 29 without PDADMAC (pH = 7.0) and (f) photographs. (g) Absorption spectra of sensor 29 with PDADMAC and (h) photographs.^59^ (i) Proposed mechanism for the sensing of cobalt by probe 30. (j) Emission (k) and absorption spectra.^60^ (l) Chemical structure and binding mode of sensor 33. (m) Changes in the absorbance of the band for probe 31 upon the addition of different metal ions. (n) Colour change and (o) fluorescence spectra with different metals.^61^ (p) Schematic illustration of proposed sensing mechanism for 32-Co^2+^. (q) Fluorescence spectra.^62^

Hai-Bo and co-workers reported the preparation of probe 28 by mixing terpyridine and DHAB (Fig. 8a). The sensing behaviour of sensor 28 with cobalt ions in ethyl alcohol (pH = 7) was measured through titration experiments. When 0–1.0 equivalent of cobalt ions was added, a band appeared at 537 nm and the colour of the solution turned from pink to yellow. Sensor 30 exhibited peaks at 340 nm and 355 nm due to the Tpy moiety the (Fig. 8b and c). Complete fluorescence quenching occurred when 1 mole equivalent of cobalt ions was added.^58^

Naked-eye colorimeter sensor probe 29 (Fig. 8d) was also reported for the detection of Co^2+^ ions at neutral pH in pure aqueous solution. The absorption spectrum of probe 31 was measured in 10 nM HEPES buffer solution. The sensing behaviour of probe 31 was observed while adding chloride salts of different metals in the presence of PDADMAC at neutral pH. These studies were carried out in HEPES buffer solution. The absorbance intensity decreased when PDADMAC was added to 5-Br-PAPS and the peak at 447 nm shifted to 426 nm (Fig. 8e and f). The electrostatic interaction between the SO_4_^2−^ and NH_3_^+^ groups of 5-Br-PAPS and PDADMAC induced aggregation, which played a role in the detection of cobalt ions. When cobalt ions were added to the aggregation, a bathochromic shift from 425 to 635 nm was observed. The LOD was calculated to be 0.51 μmol L^−1^.^59^ Gaurav Bartwal reported the preparation of probe 30 (Fig. 8i) by the diazotization of ampyrone, followed by addition of a solution of 6-hydroxyquinoline. Characterization was performed using FT-IR, H-NMR and H-RMS. A standard solution of the probe was prepared in MeOH : H_2_O and HEPES buffer solution was used to prepare the salt solution of metal ions. These solutions were diluted before the experiments. The amount of water was increased to make a water and methanol 1 : 1 solution but no significant change was detected in the emission spectra. The emission intensities of the probe must be kept nearly constant, which cancelled the AIEE characteristics (aggregation-induced emission enhancement) of the probe. The solubility of the probe decreased on increasing the amount of water and precipitation occurred, which affected the measurements and the sensitivity of the probe. Consequently, during all the photophysical measurements, the methanol to water ratio kept at 1 : 1. Different cations were added, but only cobalt ions caused the disappearance of the peak at 253 nm with a new band emerging at 469 nm (Fig. 10k). The isosbestic point was found to be at 417 nm, which strongly suggested the existence of equilibrium between the complex and probe. Sensor 30 showed turn-on fluorescence around 500 nm when cobalt ions were added to the solution and a colour change from pale to dark-yellow was observed (Fig. 8j).^60^ Lin Wang and colleagues developed chemosensor 31 using oxadiazole as the starting emitting material, which worked as a detector for cobalt ions (Fig. 8l). They synthesized oxadiazole derivative probe 33 and its structure was characterized using spectroscopic techniques such as H-NMR and C-NMR. The aldehydic and picolinate groups on the two sides of probes made it asymmetric, which assisted in the detection of cobalt ions. This probe showed good sensitivity towards the quantitative detection of cobalt ions in real water samples. Furthermore, this sensor could also identify copper ions *via* a colour change (Fig. 8n). The probe was synthesized by stirring picolinic acid and potassium chromate in dimethylformamide at 25 °C for 30 min. The stock solution of probe 31 was prepared in acetonitrile and the solutions of metal ions were prepared in the distilled water using different inorganic salts. The titration solutions were used to determine the selectivity for Co^2+^ ions. The absorption band at 590 nm confirmed the presence of Co^2+^ ions in the solution (Fig. 10m).^61^ Another probe 32 was synthesized *via* the coupling reaction among dibromo, benzothiadiazole and benzene. The fluorescence and UV-vis spectra were recorded in different solvents such as ethyl alcohol and chloromethane to examine the effects of the structure of the solvents on the optical properties of the chemosensor. The fluorescence behaviour of probe 32 towards cobalt ions was observed when 32 was dissolved in a solution of benzonitrile or ethyl alcohol (Fig. 8p). Probe 32 was used for the detection of metals at the excitation wavelength of 350 nm. The addition of cobalt ions quenched the intensity of the band of 32 at 474 nm (Fig. 8q). It was found that the benzothiadiazole group of 32 helped in the formation of a complex with cobalt ions. The Job's plot studies showed that chemo sensor 32 exhibited a 1 : 1 stoichiometric. The nitrogen atoms present in the benzimidazole group and oxygen atoms in the phenyl group helped the chelation of probe 32 with metal ions. The binding constant obtained from the plot was 2.60 × 10^5^ M^−1^ for cobalt ions.^62^

**Fig. 9:**
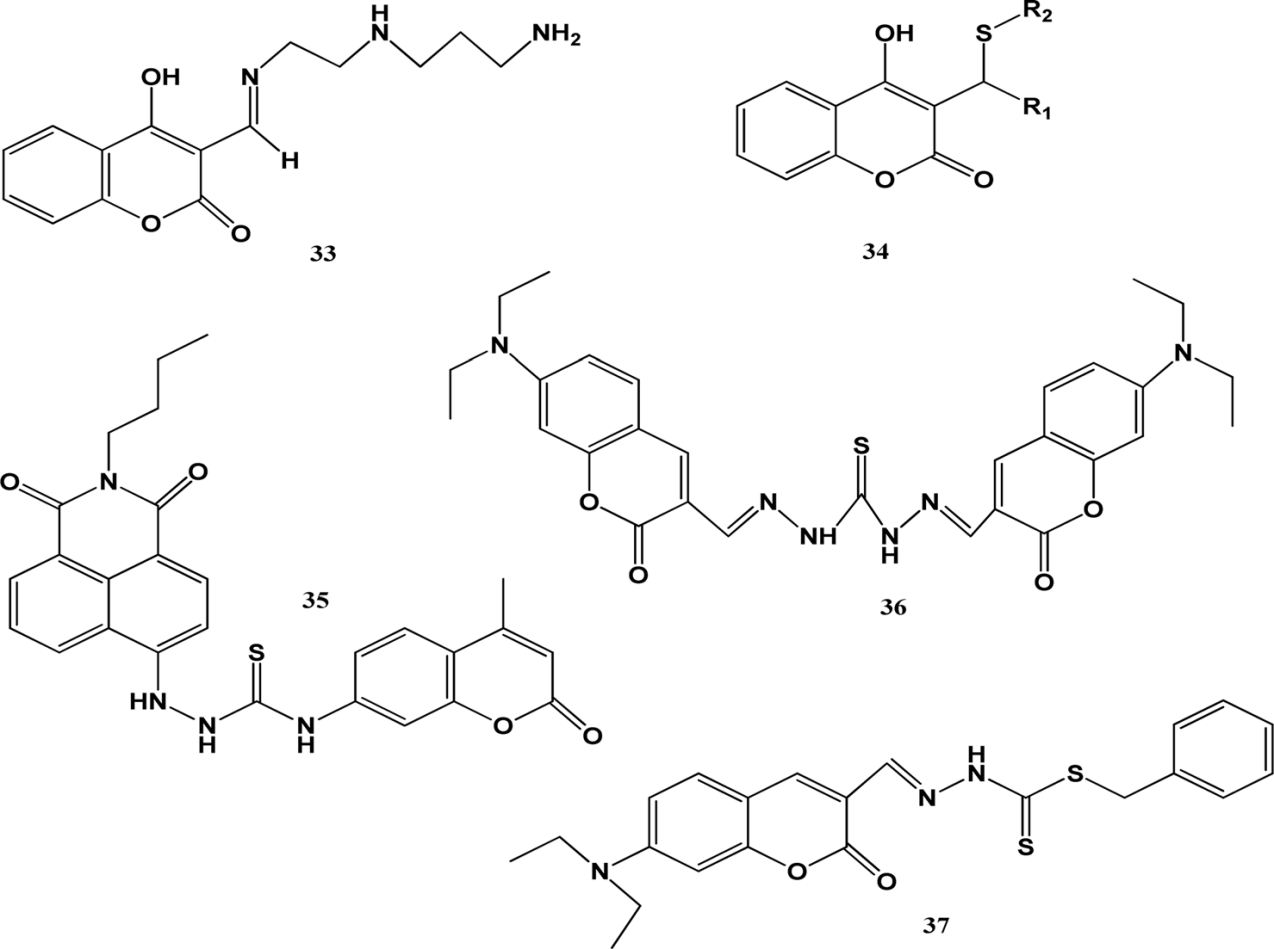
Chemical structures of chemosensors 35–39.

**Fig. 10:**
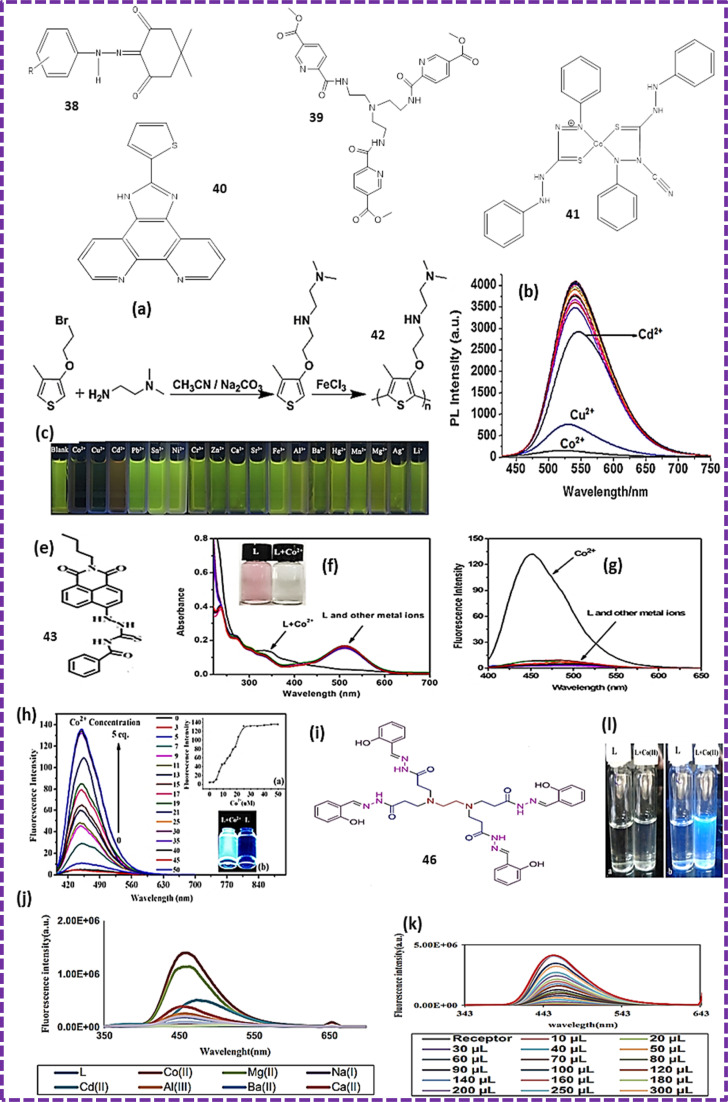
(a) Synthetic route for probe 42. (b) Fluorescence spectra of 42 with various metal ions. (c) Colour change under UV light.^72^ (e) Structure of probe 43. (f) UV-visible spectra of ligand and cobalt complex. (g and h) Fluorescence spectra.^73^ (i) Structure of chemosensor 44. (j) Change in the intensity of the fluorescence emission of the probe after adding different metals. (l) Changes in the intensity of the probe on the addition of cobalt ion solution with a gradual increase in the concentration of the solution under ultraviolet light before and after the addition of cobalt(ii) ions. (k) Study of ligand and its complex under ultra-violet light.^13^

Studies have shown that azo compounds with two phenyl groups attached to each other through a nitrogen–nitrogen double bond are very useful as chemosensors for the detection of cobalt ions. The properties of the azo compounds can be tuned by the addition of special functional groups. The presence of nitrogen and oxygen atoms in the phenyl group are responsible for the chelation with cobalt ions. Azo chemosensors have been studied due to their colorimetric response with cobalt ions at the commercial level. The real sample analysis for the search of cobalt ions is facilitated by the production of instant test kits with high sensitivity. However, a much work is required to develop azo-based chemosensors that not only detect cobalt metal ions but are even capable of removing them from the targeted region, where there is still a long way to go. These chemosensors will have a large number of applications related to living systems and the environment (Table 4).

**Table 4:** Azo and diazo compounds for the detection of cobalt ions

Probe	Medium	Ex/Em (nm)	Association constant Ka/M	Limit of detection	Application	Ref.
28	EtOH HEPES buffer solution (pH = 7.0)	394/537	7.45 × 10^5^	0.45 μM	Probe 30 assisted in the identification of cobalt ions using a commercially available reagent	58
29	HEPES buffer (pH 7.0)	425/586	2.98 × 10^8^	0.51 μM	Selective colourimetric detection of cobalt ions in real samples	59
30	Methanol/water	400/548	4 × 10^6^	10^−8^ M	Probe 32 helped in the visual inspection of metal ions in a highly alkaline environment. It changed the colour of the probe from yellow to colourless	60
	HEPES buffer (pH = 7.2)					
31	CH_3_CN/DMF	280/350	5.3 × 10^2^	3.92 μM	Probe 33 could be used for the production of instant test kits for detecting cobalt ions in real samples with high accuracy	61
32	Ethanol and CH_3_Cl	350/474	2.60 × 10^5^	41 μM	Probe 34 showed great selectivity towards cobalt ions due to its high quantum yield. It could be used for the synthesis of test kits	62

### 2.5. Coumarin compounds

Coumarins have good stability in light, large Stokes shift and low toxicity, which make them a good precursor for chemosensors. Mostly, these chemosensors are developed to contain –NH_2_ or –OH groups at 7-position and an acetyl moiety located at the 3-position. Some coumarin-based chemosensors are weakly fluorescent due to PET, whereas others are highly fluorescent. However, their interaction with metal ions reverses their fluorescence property. Coumarins contain 2 rings but the C=C bond is fixed, which traps them in the *cis*-conformation and avoids conformational changes, increasing their fluorescence emission and photostability. Numerous coumarins function properly in non-aqueous medium, which hinders the complete biocompatibility of most coumarin-based chemosensors.

Devika and co-workers prepared probe 33 based on the coumarin skeleton, which was used for the colorimetric detection of cobalt ions. The ligand was dissolved in 1 : 9 v/v ethanol : water and its UV-visible absorption investigated. The π–π* transitions of the alternating double and single bonds of the aromatic-ring were responsible for the peaks at 240 nm and 302 nm because of the less energetic n–π* transitions. The emission at 445 nm was attributed to photo-induced electron transfer (PET) and the colour of the solution changed to brown on the addition of cobalt ions. The results of the FT-IR analysis verified that the stoichiometric ratio of the complex between probe 33 and cobalt ions was 1 : 1. This probe had an efficient property to detect Co^2+^ ions from real water samples, which were collected from various places. Gaussian 3 was used to carry out DFT calculations to determine the binding mode of the ligand with cobalt ions. Structure optimization revealed the formation of the keto form. The high negative energy of the optimized structure confirmed the formation of a stable metal–ligand complex. The theoretical spectrum superimposed with the experimental results confirmed the formation of a 1 : 1 stoichiometric complex.^63^ A derivative of 4-hydroxy-3-thiomethylcoumarin was synthesized with the reaction of aldehydes, 4-hydroxycoumarin and thiol in the presence of l-proline catalyst in C_2_H_5_OH, yielding probe 34 having selectivity towards cobalt ions. The ligand showed fluorescence turn-off property when cobalt ions were added to its solution. Fluorescence quenching was observed at approximately 80% in 1 : 9 HEPES/DMSO when the pH was 7.4 and concentration was 10 mM. In UV-visible spectrum, significant changes occurred with the obvious development of isosbestic points, showing the formation of a cobalt ion and ligand complex. Two absorption bands appeared at 254 nm and 313 nm. Emission bands were produced at 401 nm when excited at 320 nm. The Job's plot confirmed the binding ratio of 2 : 1 and the binding constant was calculated to be 9.3 × 10^4^ M^−1^. This ligand was found to be the best tool for the detection of both Co and Ni ions in an appropriate environment.^64^ Liguo and co-workers reported the integration of coumarin, naphthalimide fluorophore, and thiourea functional group to prepare a probe. The medium used for the preparation of the stock solutions of the ligand was CH_3_CN : HEPES (7 : 3 volume by volume). The pH range for the analysis was 7.2 to 7.4. Changes in the fluorescence intensity of chemosensor 35 were observed with 5 equivalents of 10 μM solutions of cobalt ions. The emission peak appeared at 425 nm, which was enhanced 120 times. The LOD was found to be 6.0 nM. The UV-vis and fluorescence spectroscopic techniques were applied to confirm the formation of a ligand–metal complex. The main application of probe 36 was the detection of cobalt ions in HeLa cells.^65^ Chemosensor 38 based on a coumarin ring was synthesised. An absorption band was observed at 470 nm when this probe was analysed by the ultraviolet-visible spectroscopic technique The pH range of 2.0 to 12.5 was found to be the best working range for probe 38. A small blue-shift and slight quenching in the intensity of probe occurred in the presence of a few transition metals. Alternatively, a slight red shift was observed in the band when treated with some other ions but there was no colour change observed except for Co^2+^ ions. The absorption band of probe 36 in the spectrum showed a red shift from 490 to 510 nm when treated with a cobalt ion solution. The red shift is associated with other prominent effects, which helped decide the presence of relevant metal ions. The cobalt ions changed the yellow characteristic colour of probe 36 to deep pink, which helped the colorimetric detection together with the quenching of absorption intensity. The Job's plot confirmed that the stoichiometric ratio was 2 : 1. Chemosensor 36 was used to make kits to carry out colorimetric detection and as a staining agent for microorganisms.^66^ Another coumarin-based ligand 37 was synthesized by reacting 7-(*N*,*N*-diethylamino) coumarin-3-aldehyde with *S*-benzyl dithiocarbazate in ethanol. CH_3_CN–water was used as the solvent for the investigation of probe 37 through the UV-visible spectroscopic technique. Probe 37 alone showed absorption at 473 nm. When the ligand was combined with cobalt with a binding ratio of 2 : 1, it showed an absorption band at 517 nm (Fig. 9).

X-ray analysis was performed to confirm the structural features of the complex. The calculated association constant was 6.91 M^−1^. The maximum absorption intensity in the presence of cobalt was found in the pH range of 5.5–10.5. The absorbance ratio reached the saturation point by adding a total of 0.5 equiv. of cobalt. IR studies revealed that the keto, imine, and thio-keto groups provided chelating sites to bind with cobalt. A prominent colour change (red) for cobalt was observed and the detection limit for the cobalt by probe 39 was found to be 0.31 μM.^67^ This study showed that coumarin compounds can be used for the fluorimetric and colorimetric detection of cobalt ions in real water samples. Their application in living systems has exhibited appreciable results but the binding constant of cobalt metal ions to coumarin compounds limits rapid detection. Derivates of coumarin compounds with simple structures are the best candidates as probes for the detection of cobalt ions in living systems due to their cell membrane permeability, biocompatibility and greater penetration into tissues and their derivatives show a very low signal to noise ratio and they produce images with high resolution. Coumarins possess pharmacological properties but they are acutely toxic in nature, which limit their applications in living cells. Thus, coumarin-based chemosensors can be extensively used in the future if work is done to screen their toxicity and improve their biocompatibility (Table 5).

**Table 5:** Coumarin compounds for the detection of cobalt ions

Probe	Medium	Ex/Em (nm)	Association constant Ka/M	Limit of detection	Application	Ref.
33	Ethanol and DMSO, DMF	240/445	2.9 × 10^4^	7.06 μM	Real samples collected from different locations were used to carry out the detection of cobalt ions using probe 35, which showed excellent quality response towards the detection of metal ions	63
34	9 : 1 DMSO/HEPES buffer, pH 7.4, 10 mM	320/401	9.3 × 10^4^	0.22 μM	Ligand 41 can be used as an effective fluorescence tool to detect cobalt and nickel in the environment	64
35	CH_3_CN (pH 7)	327/425	—	6.0 nM	Cell imaging and use of probe to detect cobalt ions in living HeLa cells	65
36	6 : 4 50 mM/HEPES MeCN	470 to 510	7.95	1.0 μM	Probe 38 was used to make kits for the colourimetric detection of cobalt ions. It was used as a staining agent in microorganisms for producing microscopic images	66
37	CH_3_CN–water	473 and 517 nm	6.91	0.31 μM	Detection of cobalt using probe 39 in aqueous solution	67

### 2.6. Other organic compounds

Annamalai and co-workers synthesized chemosensors 38^1^ and 38^2^ by the addition of NaNO_2_ to anilines, followed by the addition of dimedone and sodium acetate. Both probes were characterized by X-ray diffraction analysis and other spectrometric techniques. Cobalt ions caused changes in the morphology of the ligands. The UV-visible spectra of the chemo sensors were recorded in different solvents, and then CH_3_CH_2_OH–H_2_O (4 : 1, v/v) was selected for further analysis. A bathochromic shift was observed by increasing the polarity of the solvents. When cobalt ions were added to the yellow-coloured solution of the chemosensors, they increased the band intensity at 490 and 548 nm. Ni^2+^ ions had little effect on the emission intensity of both chemosensors. The limit of detection for the chemosensors was calculated to be in the range of 3 μm to 7 μm. The metal ions were released by the chemosensor on the addition of EDTA.^68^ The HOMO and LUMO are the molecular orbitals that are responsible for the stability of the molecules. The HOMO–LUMO transition caused the transfer of electron density from the nitro group to –OCH_3_ in the ligand. The HOMO–LUMO gap in the ligand was 3.37 eV. The molecular chemical stability is decided by the hardness and whether the ligand under investigation is a hard molecule. Molecular electrostatic potential maps were calculated to predict the reactive electrophilic and nucleophilic sites. The results indicated that hydrogen atoms provide the strongest attraction sites and oxygen atoms provide the strongest repulsion sites. Chemosensor 39 was synthesized by reacting chloro-formylation with 5-(methoxycarbonyl) picolinic under reflux for 24 h in THF. The absorption spectrum of chemosensor 41 showed two peaks at 310 nm and 370 nm in the presence of Co^2+^ ions. This study showed that the chemosensor formed a 1 : 1 complex with cobalt ions. Sensor 41 contained six peripheral atoms of nitrogen (N_2_) and one central N_2_ atom, which helped in the formation of the complex with the Co–N bond length in the range of 1.923(2)–1.945(2) Å. When the complex was treated with anions of weak acids such as CO_3_^2−^, Ac^−^, HCO_3_^−^, SO_3_^2−^, and PO_4_^3−^, its absorption intensity changed. The HNMR studies of the complex with anions showed high field shifts due to the strong interactions between the ligand and anions.^69^ Phenanthroline-based chemo sensor 40 was also prepared and well characterized. The detection of metal ions was performed in DMF, which work in buffered condition with pH in the range of 7–8. The fluorescence studies of the ligand showed emission bands at 336 nm and 442 nm upon excitation at the wavelength of 298 nm. When K^+^ ions were added to the solution of chemosensor, the intensity of the band at 336 nm was slightly quenched and that at 442 nm was significantly enhanced. However, complete quenching of the intensity of the bands occurred in the spectrum at 442 nm upon interaction with cobalt ions. Two absorption bands appeared at 308 and 336 nm in the UV-visible analysis of the chemosensor. The addition of K^+^ ions to a solution of chemosensor 40 increased the band intensity and caused a slight blue shift (Fig. 10d). The analysis revealed the formation of a 1 : 1 complex. When Co^2+^ ions were added to a solution of the chemosensor, they caused a slight red shift with an increase in intensity up to two equivalents, which confirmed the formation of a 2 : 1 complex.^70^ Chromogenic sensor 41, namely dithizone (DTZ), was reported for the distinction of Co^2+^ and CN^−^ ions in DMSO/H_2_O from other metals. The UV visible spectrum of DTZ consists of two bands at 475 nm and 610 nm, causing the solution to appear green in colour. When Co^2+^ ions were added to a solution of chemosensor 41, they produced a penta-heterocycle chelating complex, which changed the colour of the solution to red. Metal ions also caused a bathochromic shift from 475 to 482 nm with the quenching of the intensity of both bands. The nitrogen and sulphur atoms of 41 behaved as donor atoms in the formation of the complex. A 2 : 1 stoichiometric complex was confirmed by the Job's plot analysis. There a small interference was observed in the presence of a few other metals in the detection process. The [Co(DTZ)_2_]^2+^ complex was used for the detection of CN^−^ ions. CN^−^ ions when introduced to a solution of the complex in DMSO caused a blue shift from 482 to 475 nm and a decrease in absorbance intensity at 610 nm. It changed the colour of the solution to orange. The detection of CN^−^ ions was interrupted by AcO^−^ and BzO^−^ ions. The selectivity studies showed that water affected the reactivity of the complex with anions. 25 vol% water in DMSO made the solution selective for the detection of CN^−^ ions and it produced a 2 : 1 complex. The response time of chemosensor 41 towards Co^2+^ ions and complex towards CN^−^ ions was 30 s. The pH did not change during the study. FTIR and HNMR spectra were used to study the structure of the complexes.^71^

Probe 42 was synthesized by suspending anhydrous ferric chloride in dry chloroform (Fig. 10a). Precipitation of the polymer resulted when the reaction mixture was treated with methyl alcohol, and then ammonium hydroxide solution was used to de-dope the polymer solution. The final polymer 42 was obtained in the form of a dark-red solid. The probe was characterized by FTIR, GPC and HNMR. The HNMR spectrum of the probe showed the disappearance of peaks and proved the removal of two hydrogen atoms, indicating the successful synthesis of the probe. With a very low level of cobalt ions, quenching of the photoluminescence was observed and the fluorescence intensity decreased rapidly at an elevated concentration of cobalt ions, which caused a slight hypochromic shift due to the de-conjugation effect on the backbone of the polymer. PL showed quenching at 534 nm. The quenching tendency was not sustained after the adding of 1 × 10^−4^ mol L^−1^ Co^2+^ and at this concentration, about 97% fluorescence of the probe was quenched (Fig. 10b). The absorption band of chemosensor 42 gradually showed quenching at 420 nm and there was an increase in intensity at 472 nm in the presence of cobalt ions. The isosbestic point was obtained at 369 nm for cobalt ions, which indicated the complexation equilibrium between the sensor and cation. The results revealed the sensitivity and selectivity of probe 42 towards metal ions. It has potential applications under biological and environmental conditions.^72^ A selective fluorescent probe 43 was synthesized from 1,8-naphthalimide derivative (Fig. 10d), which showed a weak broadened emission spectrum corresponding to ICT (intramolecular charge transfer) transition. Then, 5 equivalents of ions of different metals were added to a solution of ligand 43, which showed no or small turn-on fluorescence spectrum except in the presence of cobalt ions, showing a prominent enhancement in fluorescence intensity (Fig. 10f). Cobalt ions resulted in a prominent increase in the emission intensity of 43. Detection was also possible by the naked-eye because of the prominent colour change. Cobalt ions immediately caused the disappearance of the pink colour of 43 and made the solution colourless (Fig. 10e). The ligand metal complex showed an enhancement in the intensity of the fluorescence of ligand solution prepared in CH_3_CN and HEPES in a 4 : 1 ratio (v/v) and pH of 7.4. The determined LOD was 0.26 μM. The Job's plot and UV-visible analysis certified a 1 : 1 complex stoichiometry, while the association or binding constant value was 1.2 × 10^4^ M^−2^. Probe 43 was found to be a good sensor for the detection of cobalt in the biological field, showing less harmful properties in organisms and fine cell permeability in cell imaging.^73^ Li's team made CDs from cysteine through the reaction of cysteine molecules and cobalt ions. However, this type of identification resists the reactive raw materials of CDs. Fluorescent probe 44 was prepared by the reaction of diethylenetriamine and Carbopol, which were dissolved in deionized H_2_O at 200 °C for 5 h in a poly autoclave. The absorption band was produced by the probe at 320 nm, while an emission was observed at 430 nm when excited at a wavelength of 340 nm. When cobalt ions were added to a solution of sensor 44, the colourless solution changed to brown.

The colour change in the solution enabled the colorimetric detection after the binding of cobalt ions with ligand 44. The fluorescence intensity was quenched when the concentration of the Co metal ions was increased and turn-off behaviour observed. The two analytical techniques used for the characterization were UV-visible spectrophotometry and PL (photoluminescence) spectrophotometry. Two molecules of the receptor combined with one molecule of Co metal ion, producing a 2 : 1 stoichiometric complex. The determined limit of detection (LOD) was 0.45 μM.^74^ Shenyi and co-workers synthesized probe 45 from pyridine and naphthalene derivatives. Probe 45 showed an absorption peak at 457 and emission peak at 528 nm. In the presence of cobalt ions, the emission peak shifted to 474 due to internal charge transfer (ICT). The coordination of sensor 45 with Co^2+^ was also investigated by minimum energy molecular modeling. The cobalt ions are coordinated by the nitrogen atoms (four) of the two aminomethyl pyridines, in which the amino group coordinates with the cobalt ion and ICT occurs. Cobalt metal ions resulted in a prominent enhancement in the peak. Job's analysis proved 1 : 1 binding. The binding or association constant was 1.1 × 10^7^ M^−2^. This fluorescent probe 45 was used for to detect cobalt *in vitro*.^75^ Another receptor 46 with dendritic macromolecule was synthesized by the reaction of 1 mole of PAMAM and 4 moles of hydrazine hydrate without stirring the solvent for 4 h at room temperature (Fig. 10i). The probe showed turn-on behaviour with an increase in cobalt metal ions (Fig. 10k). The stoichiometric ratio of the ligand and the cobalt was 1 : 2, which was confirmed by two techniques, *i.e.* mass spectroscopy and Job's plot technique. The maximum emission was observed at 460 nm and the LOD was 32.3 nM. The complexation of the dendritic chemosensor with cobalt metal ions was characterized by several different analytical techniques including FTIR spectroscopy, FT Raman micro-spectroscopy, fluorescence and UV-vis spectroscopy, ^1^HNMR and ^13^CNMR (Table 6).^13^

**Table 6:** Other compounds for the detection of cobalt ions

Probe	Medium	Ex/Em (nm)	Limit of detection	Application	Ref.
38	Ethanol/water	490 and 548	3 μm to 7 μm	It is used to make sensor for the colourimetric detection of cobalt ions	68
39	DMSO–H_2_O (50/1, v/v)	310/310 and 370	10 μM	Probe 39 was used to synthesize colourimetric chemosensor for cobalt metal ions and for anions of weak acids	69
40	DMF solution that buffered by 0.1 mM NaOAc–HOA with working pH from 7 to 8	Emission bands at 336 and 442	100 μM	Probe 40 was used for the development of a ratiometric chemical sensor for the detection of cobalt and potassium ions	70
41	DMSO/H_2_O in 4 : 1	Ligand: 475/610	0.04 μM	Detection of Co^2+^ and CN^−^ ions in real samples	71
		Complex: 482/610			
42	MeCN/Tris–HCl solution	412/534	2.5 nM	Detection of cobalt ions in real samples	72
43	CH_3_CN, HEPES buffer, pH 7.4	380/450	0.26 μM	Probe 43 can act as chemosensor for the detection of cobalt ions in the biological environment due to its less toxic behaviour towards living organisms and used for the imaging of living cells	73
44	De-ionized water, tetrafluoroethylene Na_2_CO_3_	340/430	0.45 μM	CDs (probe 44) utilized for monitoring natural water quality for Co^2+^ contents	74
45	DMF, HEPES/EtOH (v/v: 60/40) buffer	440/528	1.0 μM	Used for the measurement of Co^2+^ both *in vitro* and in living cells (HeLa cells)	75
46	Ethanol, DMSO	460	32.3 nM	Detection of cobalt ions	13

A quinoxaline-hydrazinobenzothiazole sensor enabled the colorimetric detection of cobalt ions. The yellow solution turned to pale brown in the presence of cobalt ions. The LOD was found to be 9.92 × 10^−8^ M. This sensor was used in daylight for naked-eye detection.^76^ Another sensor, 4-((9*H*-purin-6-yl)diazenyl)-6-hexylbenzene-1,3-diol was also reported for the colorimetric detection of cobalt ions. With the incremental addition of cobalt ions, the colour of the solution changed gradually from pale-orange to deep-red. This sensor enabled real-time sensing and good reproducibility. It showed excellent detection and quantification limit of 0.3 ppb and 1 ppb, respectively. DFT calculations corroborated the binding mechanism.^77^ Another quinoline-based sensor was synthesized for the colorimetric detection of cobalt(ii) ions. The colourless solution of the sensor changed to yellow.^78^ A hyperbranched polyethylenimine (HPEI) polymer also showed a colorimetric response upon treatment with cobalt(ii) ions across a wide pH range 4–10. Whatman filter paper strips were designed for the rapid and on-site sensing of the target metal ions.^79^

Chemosensors have made the visualization of living cells within or outside living organisms possible. They provide opportunities to study cells, organelles and even tissues by interacting with metal ions located inside these living bodies. Probes are designed to minimize the toxicity to living cells and enhance their ability to penetrate the cell membrane to reach the target organelles or a specific region inside the cell. When these chemosensors interact with metal ions in these bodies, they change their fluorescence properties and colour, which help in their visualization.^80^ Chemosensor 46 was found to be less toxic and used by Yu-Long Liu and colleagues for the cell imaging of HepG2 cells. They incubated these cells with 48, and then treated them with Co^2+^ ions, which survived for 24 h, and their images recorded. They merged the fluorescent and bright field images, which provided good images with visualization of the intracellular regions of the cells.^15^ Scientists have extensively searched for probes with biocompatibility and the least cytotoxicity. They can help scientists understand the cellular mechanism in great depth without disrupting the normal functioning of cells. Chemosensors are also used to study essential cellular bodies such as mitochondria and endoplasmic reticulum and biomolecules through imaging. Abnormal functioning of these entities can lead to fatal consequences, and thus fluorescence imaging investigation provides a tool to understand and develop cures against a variety of related problems. For instance, a pyrene-based peptide beacon was used to change the conformation of DNA and used in the analysis of nucleic acids.^81^ A ligand-directed tosyl-containing coumarin fluorophore was used to study the targeted proteins. When it was attached to the protein surface, the probe underwent cleavage, causing the fluorophore and quencher to separate. However, the addition of analyte recovered the fluorescence of the probe and used for the study of target proteins.^82^ However, although a variety of work has already done to improve the biocompatibility and reduce the toxicity of chemosensors to make them available for use in the treatment of human beings, there is a long way to achieve this goal.

## 3. Nanomaterials for cobalt sensing/detection

There are numerous alternative methods for the quantitative determination of metal ions and the researchers have focused on the development of facile and cost effective techniques. Various nanoparticles (NPs) have been employed for detection of cobalt ions. Sung *et al.* reported the preparation of glutathione (GSH)-modified silver nanoparticles (AgNP) with different shapes for the selective colorimetric trace analysis of cobalt(ii) ions. Rod-type GSH-AgNP showed high sensitivity towards cobalt(ii) ions.^83^*N*-Cholyl-I-valine(NaValC)-based AuNPs were also reported for the colorimetric detection of Co^2+^ and Ni^2+^ in environmental samples. The selectivity was dependent on sunlight irradiation, pH of the solution medium and reaction time. This sensor was proven to be economical and eco-friendly.^84^ Karami *et al.* also reported the preparation of gold nanoparticles (AuNPs) from glycyrrhizic acid (GA). The detection limit for cobalt ions was determined to be 0.4 nM.^85^ AgNPs are easy to prepare and have surface plasmon resonance (SPR) bands, which help in simple analysis by Ultraviolet-visible (UV-vis) spectroscopy.^86^ A specific class of colorimetric transducer gold nanoparticles (Au-NPs) exhibited intense red colour as a result of SPR absorbance. Au-NPs were used as a template to anchor calixarene and treated with cobalt(ii). A distinct colour change from pink to blue was observed, which was even noticeable by the naked-eye. The detection limit was reported to be 10^−9^ M with no interference with other metal ions.^87^ Rapid colorimetric sensing by plasmonic nanoparticles has attracted wide attention due to its simplicity and low cost. Novel optical probes based on Ag–Au bimetallic nanoparticles were reported for the selective determination of cobalt(ii) ions in the presence of other ions (Fe^3+^, Fe^2+^, Zn^2+^, Pb^2+^, Cu^2+^, Ni^2+^, Ag^+^, Mn^2+^, Hg^2+^, K^+^ and Ba^2+^) with the detection of 0.02 μM metal.^88^ The presence of cobalt(ii) ions in water was investigated by silver nanoparticles, which were capped with 3-mercapto-1-propanesulfonic acid sodium salt (AgNPs-3MPS). This optical sensor showed a shift in the shape and intensity of its absorption peak due to SPR when treated with cobalt(ii) ions. A strong colorimetric effect was observed when the concentration of cobalt(ii) ions was 1 ppm (part per million) and the specific sensitivity was reported as 500 ppb (part per billion). The mechanism responsible for the detection of cobalt(ii) ions by AgNPs-3MPS was prosed as the formation of a coordination compound.^89^

Quantum dots (QDs) enable the rapid fluorescent detection of metal ions, but their colorimetric detection remains a challenging task for researchers. However, carboxyl-functionalized cadmium sulphide (COF-CdS) was reported for the colorimetric detection of cobalt(ii) ions in water. The UV-vis absorption spectrum of COF-CdS was enhanced, accompanied by a colour change from colourless to yellowish brown after 5 min. The limit of detection was reported to be 0.23 μg mL^−1^ and percentage recoveries ranged from 99.63% to 102.46%. The use of the sensor was simple, rapid and cost effective for the determination of cobalt(ii) ions in an aqueous environment.^90^ Moreover, graphene quantum dots based on nitrogen and sulphur (N,S-GQDs) were developed for the sensitive, convenient and selective determination of cobalt(ii) ions. The metal–ligand interaction between N,S-GQDs and cobalt(ii) ions quenched the fluorescence intensity. Furthermore, their aggregation enhanced their UV-vis absorption at 430 nm with a colour change from colourless to yellowish brown within 3 min with a detection limit of 1.25 μM in an aqueous environment. Furthermore, this sensor possessed low cytotoxicity and was proposed for application in the biological and environmental fields.^91^l-Cysteine-based carbon dots with nitrogen and sulphur (N,S-CDs) synthesized *via* the hydrothermal method showed an enhancement in fluorescence intensity when treated with cobalt(ii) ions in the presence of other metal ions. Rapid colorimetric monitoring with a detection limit of 26 nM was shown by the sensor. The quenched fluorescence intensity of N,S-CDs induced by cobalt(ii) ions was recovered by the addition of EDTA or H_2_O_2_. A complex was formed by N,S-CDs and cobalt(ii) ions due to electron transfer, resulting in static quenching.^92^ One-pot-synthesized nitrogen and sulphur co-doped carbon dots (NS-CDs) were also reported to show a colorimetric response in the presence of cobalt(ii) ions.^93^ Also, luminescent sulphur dots (*S*_dots_) were synthesized from sodium thiosulfate, which showed a quantum yield of 2.5% under ultraviolet light irradiation. *S*_dots_ showed a colorimetric response against cobalt(ii) ions with multiple metal ions such as Cr^6+^ and Pb^2+^ ions. It was proposed that *S*_dots_ are a promising candidate for portable devices based on a single-element nanomaterial.^94^ Fluorescent silicon quantum dots (SiQDs) were reported to be a highly stable and water soluble sensor for cobalt(ii) ions with a limit of detection of 0.37 μmol L^−1^. The fluorescence intensity of the probe was quenched remarkably and the mechanism of quenching was proposed as static in nature. Test paper-based SiQDs proved to be highly sensitive, selective and cost effective, which were used to detect cobalt(ii) ions in environmental water samples.^95^ Liao et el. reported the hydrothermal synthesis of phosphorous, nitrogen co-doped carbon quantum dots (P,N-CQDs) from pyridoxal 5-phosphate and ethanediamine. This probe was highly selective for cobalt(ii) ions with a linear range of 0–60 μM and LOD of 0.053 μM. This probe also showed recovery of cobalt(ii) ions when treated with ethylenediaminetetraacetic acid-modified Fe_2_O_3_@SiO_2_.^96^ Also, fluorescent nitrogen-doped carbon dots (NCSs) were reported for the detection of cobalt (ii) ions with a linear range of 1.0–60 μM and LOD of 0.25 μM.^97^ 3-Mercaptopropoi-based cadmium sulphide quantum dots (MPA-CdS-QDs) showed a Stokes shift when interacting with cobalt(ii) ions with a detection limit of 10 nM and the colourless solution changed to yellow. This sensor was very efficient, rapid and cost effective for the determination of target ions in an aqueous environment.^98^ Li *et al.* reported the synthesis of cysteine-based sulphur quantum dots (SQDs) for the detection of heavy metals. An as-prepared colourless solution of the chemosensor was treated with cobalt(ii) ions and the colour changed to yellow due to the photoinduced electron transfer (PET) effect. The limit of detection was determined to be 0.16 μM. This chemosensor was very efficient in environmental monitoring, cell imaging, disease diagnosis and light-emitting diodes.^99^

## 4. Conclusion

In this review, the recent advancements in the fluorimetric and colorimetric detection of cobalt ions were discussed in different categories, enabling readers to have a quick overview in designing effective and efficient probes. This field is still flourishing and has attracted significant attention from researchers. The topology of existing ligands shows selective binding with specific metal ions. The chemosensors discussed in this study have a detection limit of up to 10^−9^ M but Schiff bases show the greatest sensitivity, reaching a detection limit of 10^−14^ M. The rhodamine moiety occupies a large backbone segment, which not only allows less toxicity towards living cells but also facilitates the detection of metal ions in aqueous/semi-aqueous medium. The ring opening of spirolactam to form amide is responsible for colorimetry. Moreover, the multiple bonds with heteroatoms (O, N, S and P) present in Schiff bases, azo dyes, coumarins, anthraquinone, hydrazides, *etc.* provide a cavity responsible for conjugation, resulting in colour changes. This article highlighted the declining interest by researchers towards the development of chemosensors based on coumarin and anthraquinone compared to other compounds, indicating that there is still space for designing new strategies for the development of efficient chemosensors. This review article is an addition to the existing library of extensive reports on chemosensor research.

## 5. Future perspective

In the pursuit of practical applications of various ligands for the detection of cobalt ions in the environment, rationally designed compounds may be synthesized to optimize their stability, solubility and reversibility. Recently, enthusiastic progress has been made in designing ligands for practical applications but their use on a commercial scale is still limited by problems, and some important mechanisms also need to be unearthed. The directions and key factors for the development of effective ligands are as follows:

(i) The structure of the ligands influences the binding of metal ions. The presence of electron-donating groups and donor atoms in close proximity not only affects the sensitivity but also provides multiple binding sites for metal ions.

(ii) Metal ions are detected either based on fluorescent spectroscopy or colourimetry. The majority of sensors lack colour changes and rely on excitation and emission data. Colourimetry provides on-site detection of metal ions with instant colour change, and thus the presence of a fluorophore in the ligands may be targeted to design ligands for the naked-eye detection of metal ions.

(iii) For commercial applications, the colourimetric properties of the ligands may be transformed in the form of various portable materials such as strips and kits. A change in electrochemical properties with the complexation of metal ions with ligands can be explored extensively to devise electrochemical sensors for efficient qualitative and qualitative analysis. Moreover, the development of cost-effective and eco-friendly sensors requires in-depth understanding with the correlation of experimental and computational studies.

(iv) Obviously, few researchers have reported the reversibility of the complexation in the presence of other ligands but insight into the decomplexation mechanism is necessary for the separation of metal ions on a large scale. By focusing on factors affecting the complexation and decomplexation such as solvent, pH, temperature and presence of other metal/non-metal ions, different ligands may be designed to remove cobalt ions from the environment and living things.

Despite the existing challenges, the various types of ligands discussed in this review pave the way for the development of efficient probes. Low-cost materials and eco-friendly nature of sensors guarantee their commercial applications. In this review, the ligands are categorised systematically and profound prospects were highlighted, which will provide a road map for the precise construction of new chemosensors for fluorimetric and colorimetric identification and separation of metal ions in the future.

## Conflicts of interest

The authors declare that they have no known competing financial interests or personal relationships that could have appeared to influence the work reported in this paper.

## Supplementary Material
